# Chlorpromazine affects glioblastoma bioenergetics by interfering with pyruvate kinase M2

**DOI:** 10.1038/s41419-023-06353-3

**Published:** 2023-12-13

**Authors:** Claudia Abbruzzese, Silvia Matteoni, Paola Matarrese, Michele Signore, Barbara Ascione, Elisabetta Iessi, Aymone Gurtner, Andrea Sacconi, Lucia Ricci-Vitiani, Roberto Pallini, Andrea Pace, Veronica Villani, Andrea Polo, Susan Costantini, Alfredo Budillon, Gennaro Ciliberto, Marco G. Paggi

**Affiliations:** 1 Cellular Networks and Molecular Therapeutic Targets, Proteomics Unit, IRCCS - Regina Elena National Cancer Institute, 00144 Rome, Italy; 2 Center for Gender-Specific Medicine, Istituto Superiore di Sanità, 00161 Rome, Italy; 3 RPPA Unit, Proteomics Area, Core Facilities, Istituto Superiore di Sanità, 00161 Rome, Italy; 4 SAFU Unit, IRCCS - Regina Elena National Cancer Institute, 00144 Rome, Italy; 5 The Institute of Translational Pharmacology - IFT - CNR, Rome, Italy; 6 UOSD Clinical Trial Center, Biostatistics and Bioinformatics, IRCCS - Regina Elena National Cancer Institute, 00144 Rome, Italy; 7 Department of Oncology and Molecular Medicine, Istituto Superiore di Sanità, 00161 Rome, Italy; 8 Fondazione Policlinico Universitario A. Gemelli IRCCS, Institute of Neurosurgery, Catholic University School of Medicine, 00168 Rome, Italy; 9 Neuro-Oncology, IRCCS - Regina Elena National Cancer Institute, Rome, Italy; 10 Experimental Pharmacology Unit, Laboratori di Mercogliano, Istituto Nazionale Tumori-IRCCS-Fondazione G. Pascale, 80131 Napoli, Italy; 11 Scientific Directorate, Istituto Nazionale Tumori-IRCCS-Fondazione G. Pascale, 80131 Napoli, Italy; 12 Scientific Directorate, IRCCS – Regina Elena National Cancer Institute, 00144 Rome, Italy

**Keywords:** Pharmacology, Cancer metabolism

## Abstract

Glioblastoma (GBM) is the most frequent and lethal brain tumor, whose therapeutic outcome - only partially effective with current schemes - places this disease among the unmet medical needs, and effective therapeutic approaches are urgently required. In our attempts to identify repositionable drugs in glioblastoma therapy, we identified the neuroleptic drug chlorpromazine (CPZ) as a very promising compound. Here we aimed to further unveil the mode of action of this drug. We performed a supervised recognition of the signal transduction pathways potentially influenced by CPZ via Reverse-Phase Protein microArrays (RPPA) and carried out an Activity-Based Protein Profiling (ABPP) followed by Mass Spectrometry (MS) analysis to possibly identify cellular factors targeted by the drug. Indeed, the glycolytic enzyme PKM2 was identified as one of the major targets of CPZ. Furthermore, using the Seahorse platform, we analyzed the bioenergetics changes induced by the drug. Consistent with the ability of CPZ to target PKM2, we detected relevant changes in GBM energy metabolism, possibly attributable to the drug’s ability to inhibit the oncogenic properties of PKM2. RPE-1 non-cancer neuroepithelial cells appeared less responsive to the drug. PKM2 silencing reduced the effects of CPZ. 3D modeling showed that CPZ interacts with PKM2 tetramer in the same region involved in binding other known activators. The effect of CPZ can be epitomized as an inhibition of the Warburg effect and thus malignancy in GBM cells, while sparing RPE-1 cells. These preclinical data enforce the rationale that allowed us to investigate the role of CPZ in GBM treatment in a recent multicenter Phase II clinical trial.

## Introduction

Glioblastoma (GBM) is the most common and malignant primary brain tumor in adults [1]. Even when treated using the best available therapeutic protocol, GBM is associated with a median overall survival of 14.6 months and a 5-year survival <5% [2], denoting an unmet clinical need. Frequent clinical relapses of GBM are due to (a) its highly invasive nature [3]; (b) the difficulties of surgical removal [4]; (c) the existence of different cell subsets of progenitor and glioma stem cells that resist to and adapt under therapeutic pressure [5–7]; and (d) the ability to build functional networks able to invade the surrounding parenchyma and repair, when damaged, interconnected cancer cells [8]. Therefore, novel, effective therapeutic approaches are quite difficult to design but urgently needed.

Drug repurposing, the discipline that discovers new applications for old drugs, allows effective medications to be brought from bench to bedside and appears applicable to GBM [9]. Antipsychotic drugs play an important role in this setting [10], also according to the recent identification of tumor-neuron synaptic connectivity through which GBM cells use neuromediators as oncogenic stimuli [11]. Recently, we investigated the effect of the neuroleptic drug chlorpromazine (CPZ) in inhibiting several molecular and cellular parameters in GBM cells [12, 13], thus paving the way for repurposing this drug in GBM therapy in combination with the first-line therapeutic approach described in 2005 by Stupp et al. [2].

CPZ is a safe drug listed in the 2021 WHO Model List of Essential Medicines (current version) [14]. It achieves its pharmacological effect in psychiatric disorders by non-specific interference with several CNS neurotransmitter receptors [10, 15, 16]. To delve into the mode of action of CPZ, especially as a potential anticancer drug, we undertook two proteomics approaches: (a) Reverse-Phase Protein microArrays (RPPA), to evaluate the effects of the drug on signal transduction pathways and (b) activity-based protein profiling (ABPP) followed by mass spectrometry (MS) analysis, to identify potential hitherto unknown molecular targets. Our data accurately defined the interference of CPZ in modifying major signal transduction pathways and suggested a role for CPZ in interfering with the cellular factor pyruvate kinase (PK) M2. PKM2 is a PK variant that represents a distinctive trait for many cancers and is pivotal for the orchestration of metabolic changes, epitomized as the Warburg effect [17–19].

## Results

### CPZ alters pivotal signal transduction pathways in GBM cells

The genomic complexity of GBM makes it extremely difficult to predict therapeutic vulnerabilities based only on molecular analyses at a genetic level. Indeed, at a steady state and under environmental pressures, the spectrum of genomic lesions results in the functional downstream integration of several aberrant signaling pathways in individual GBM patients. Therefore, we sought to use the RPPA to analyze the pathway-level effects of CPZ on GBM cells. To this end, we selected 49 endpoints (The list of the antibodies employed is available as raw data, see below**)**, mainly implicated in autophagy and metabolism, and measured the effects of CPZ treatment in GBM cells in vitro.

**Fig. 1:**
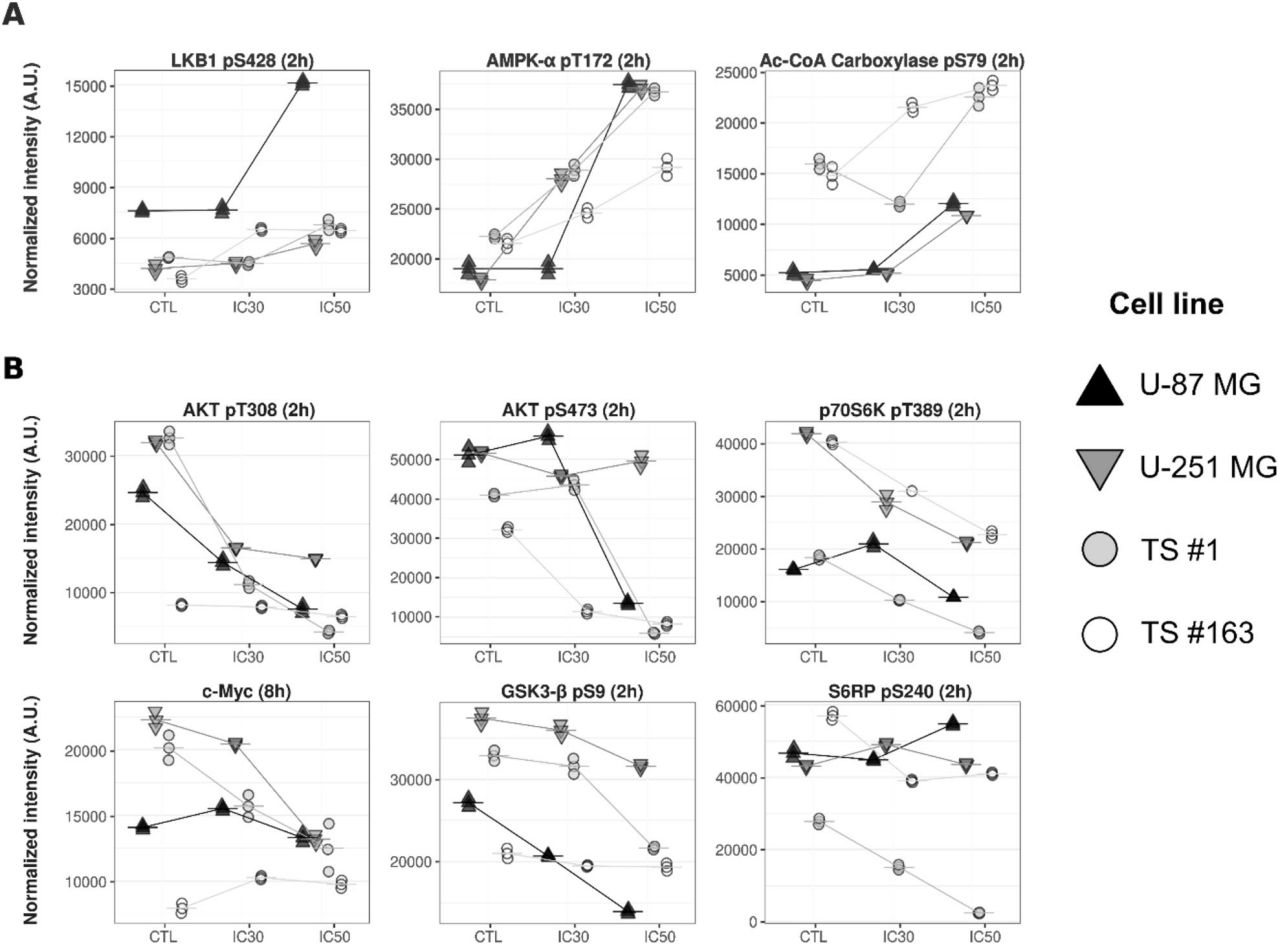
RPPA analysis of anchorage-dependent GBM cells and neurospheres challenged with CPZ. The panels include selected plots of normalized RPPA levels (Arbitrary Units, AU) for (**A**) endpoints implicated in autophagy and (**B**) PI3K-mTOR metabolic network, as measured over a three-point dose response of CPZ (Control, IC30 and IC50, from left to right) at either 2 or 8 h. *N* = 3.

Interestingly, consistent with the biological diversity of GBM, we found that CPZ treatment either hindered or fueled diverse targets in individual cell lines (Figs. 1, S1). Nonetheless, regardless of the dose or timing of the effects produced in anchorage-dependent GBM cells and neurospheres, CPZ treatment led to a constant increase of the autophagic response, i.e., increased phosphorylation of LKB1 pS428, AMPK-α pT172 and Ac-CoA Carboxylase pS79 (Fig. 1A), which appears consistent with our previous report [13]. Interestingly, in a closely related context, we found an inhibition at several levels of the PI3K/AKT/mTORC1 anabolic pathway, resulting in an early decrease of phosphorylated AKT pT308 (2 h), which ultimately lowered the levels of c-Myc (8 h). In addition, c-Myc, via c-Myc–hnRNPs, acts as a regulator of the alternative splicing of the *PKM* gene, thus regulating the PKM1/PKM2 ratio [20] (Fig. 1B). Finally, we found that several analyzed targets showed co-regulation patterns in a cell- and time-dependent manner, further demonstrating the complex signaling network scenario of GBM (Fig. S1).

### Identification of cellular factors as putative targets of CPZ in GBM

**Fig. 2:**
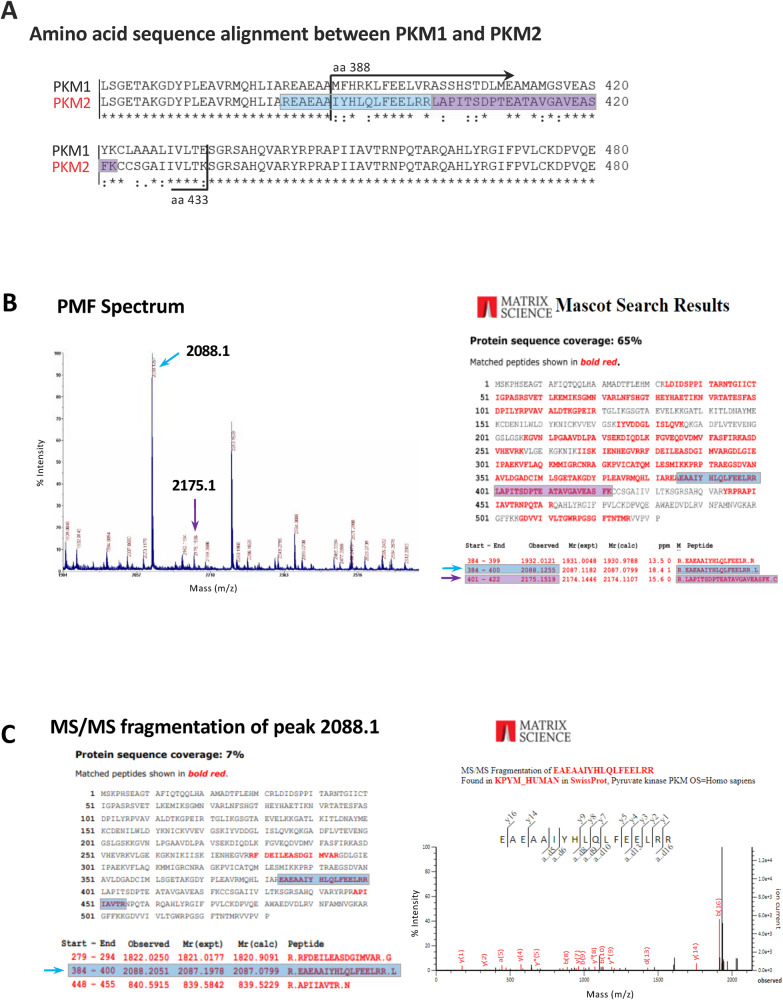
ABPP + MS output allowing the identification of PKM2 as a CPZ target. **A** Sequence alignment of PKM1 and PKM2 between aa 350 and 480. The differences between the isoforms lies within aa 388 and 433. **B** Left: a representative Peptide Mass Fingerprinting (PMF) spectrum obtained after MS analysis of the PK lane. Blue and violet arrows indicate peptides with m/z values of 2088.1 and 2175.1, corresponding to the aa sequence interval 384–400 and 401–422, respectively, distinctive of the M2 isoform. Right: the relative MASCOT PMF database identification results. **C** MS/MS fragmentation spectrum of peak 2088.1, confirming the PKM2 aa sequence.

Along with RPPA, we employed ABPP + MS to intercept cellular factors as potential direct targets of CPZ in GBM cells. Experiments conducted using a kinase enrichment procedure via an insoluble ATP probe allowed us to identify, by MS analysis, the PKM2 isoform in the U-87 MG GBM cells and TS#1 neurospheres as a factor whose binding to ATP was hindered by increasing CPZ concentrations (Fig. 2A–C).

These results uncovered a potential interference of CPZ with the PKM2 isoform of PK. To validate these results, we analyzed the effects of CPZ on several cellular and molecular processes involving PKM2.

### Interference of CPZ with GBM energy metabolism

**Fig. 3:**
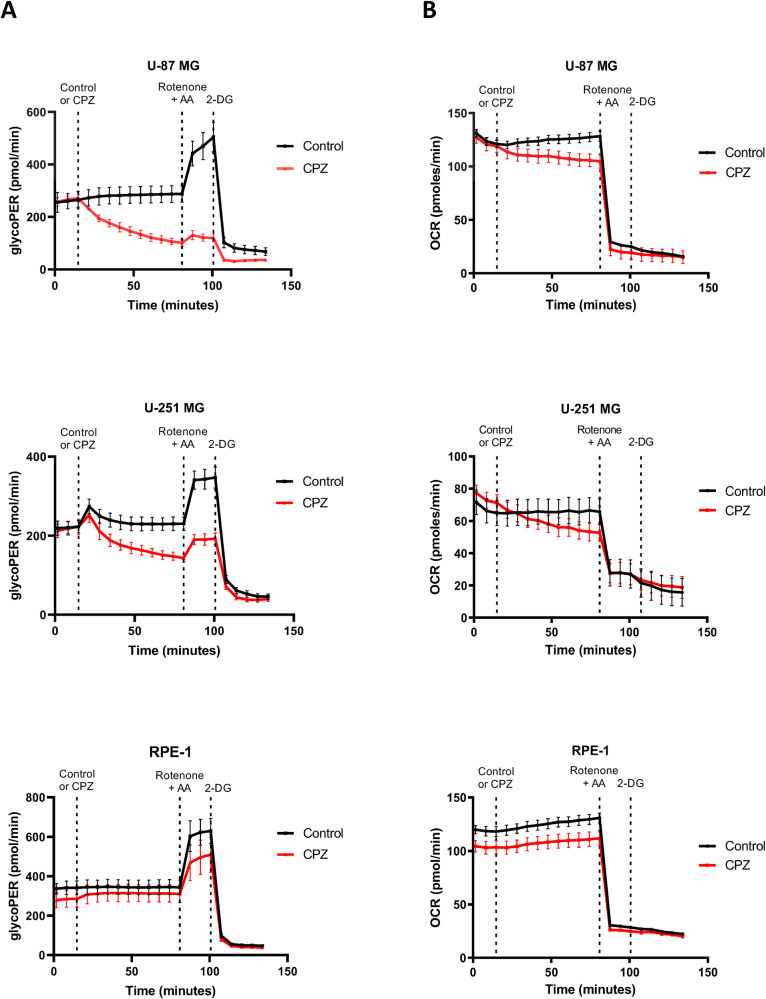
Interference of CPZ with glucose metabolism in GBM cells. Cells were incubated using the Seahorse XFp platform. Dashed vertical lines indicate, from left to right, the time of addition of CPZ or solvent for control, rotenone plus AA and 2-DG, respectively. Red lines represent CPZ-treated cells and black lines control (solvent-treated) cells. **A** GlycoPER plots related to U-87 MG, U-251 MG GBM cell lines and RPE-1 non-cancer cells. **B** OCR plots related to U-87 MG, U-251 MG GBM cell lines and RPE-1 non-cancer cells. All experiments were performed three times in triplicate. Representative graphs are shown here; dots and vertical bars indicate mean ± SD. Raw data from all experiments are available in Supplementary Material.

Since PK is a key regulatory enzyme of the glycolytic pathway, we determined the glycolytic rate in the anchorage-dependent U-87 MG and U-251 MG GBM cell lines using the glycolytic rate acute stress test [glycolytic proton efflux rate (glycoPER) and oxygen consumption rate (OCR)] performed on a Seahorse XFp platform. This technology allows real-time measurement of the derivative of the amount of extracellular lactate ions released from cells, obtained from a correction of the total H^+^ efflux, i.e., glycoPER, deriving from the Extra-Cellular Acidification Rate (ECAR) from which the value of the acidification brought about by the non-glycolytic acidification of the incubation medium has been subtracted (see Materials and Methods). Representative graphs are shown in Fig. 3. After baseline determination, cells were added sequentially with CPZ (red lines) or solvent for control (black lines), rotenone (which blocks ATP production from NADH oxidation) plus antimycin A (AA, a mitochondrial electron transport inhibitor) and, finally, with 2-deoxy-D-glucose (2-DG, a glycolytic poison). In all controls, rotenone plus AA elicited the glycolytic reserve, i.e., the glycolytic boost to compensate for the pharmacologically-induced collapse of ATP production. Finally, the addition of 2-DG dropped the glycolytic rate. As far as glycoPER was concerned, the addition of CPZ produced a significant and immediate glycolytic impairment in the U-87 MG and U-251 MG cells, further rendering them less sensitive to the glycolytic boost induced by rotenone plus AA. On the other hand, RPE-1 neuro-epithelial non-cancer cells appeared substantially less affected by CPZ (Fig. 3A). The drug also inhibited OCR in U-87 MG and U-251 MG GBM cells, while this parameter was unchanged in the RPE-1 non-cancer cells (Fig. 3B).

In the attempt to define the role of PKM2 as a target of CPZ, we performed a further experimental set, in which U-87 MG and U-251 MG GBM cells, as well as RPE-1 non cancer cells, were assayed for glycoPER and OCR after PKM2 depletion via siRNA PKM2 silencing. siRNA-PKM2-transfected U-87 MG and U-251 MG GBM cells displayed a lower effect of CPZ on these parameters, when compared with the respective controls, while PKM2 silencing in RPE-1 non-cancer cells was substantially irrelevant. Tracks refer to a representative experiment. The efficacy of PKM2 silencing via these specific reagents has been documented for these cell lines by PKM2 western blot determination in siRNA-PKM2-transfected cells when compared with the siRNA-Control counterparts (Fig. S2).

To provide a more accurate output of the role of PKM2 silencing on the effect of CPZ, we averaged the Seahorse output data from two triplicate experiments and normalized the glycoPER to 100% at the mock or drug input (Fig. S3). This revealed a differential effect of PKM2 silencing on the two GBM lines. We quantified the platform output data and displayed them as histograms to compare the differences in cell behavior in induced or compensatory glycolysis between the two GBM cell lines, in which the effect of the drug resulted apparent and partially related with PKM2 overall expression. Conversely, in the non-cancer RPE-1 cell lines, variations were apparent solely in compensatory glycolysis where it was function of PKM2 expression and independent of the treatment with CPZ.

Collectively, these extracellular lactate determinations show that CPZ interferes with glycoPER and OCR in GBM cells, while affecting the RPE-1 cells to a lesser extent. On the other hand, the siRNA results were less clear-cut, possibly due to the incomplete, but reproducible, PKM2 silencing achieved via specific siRNA.

### CPZ increases intracellular pyruvate amount in GBM cells

**Fig. 4:**
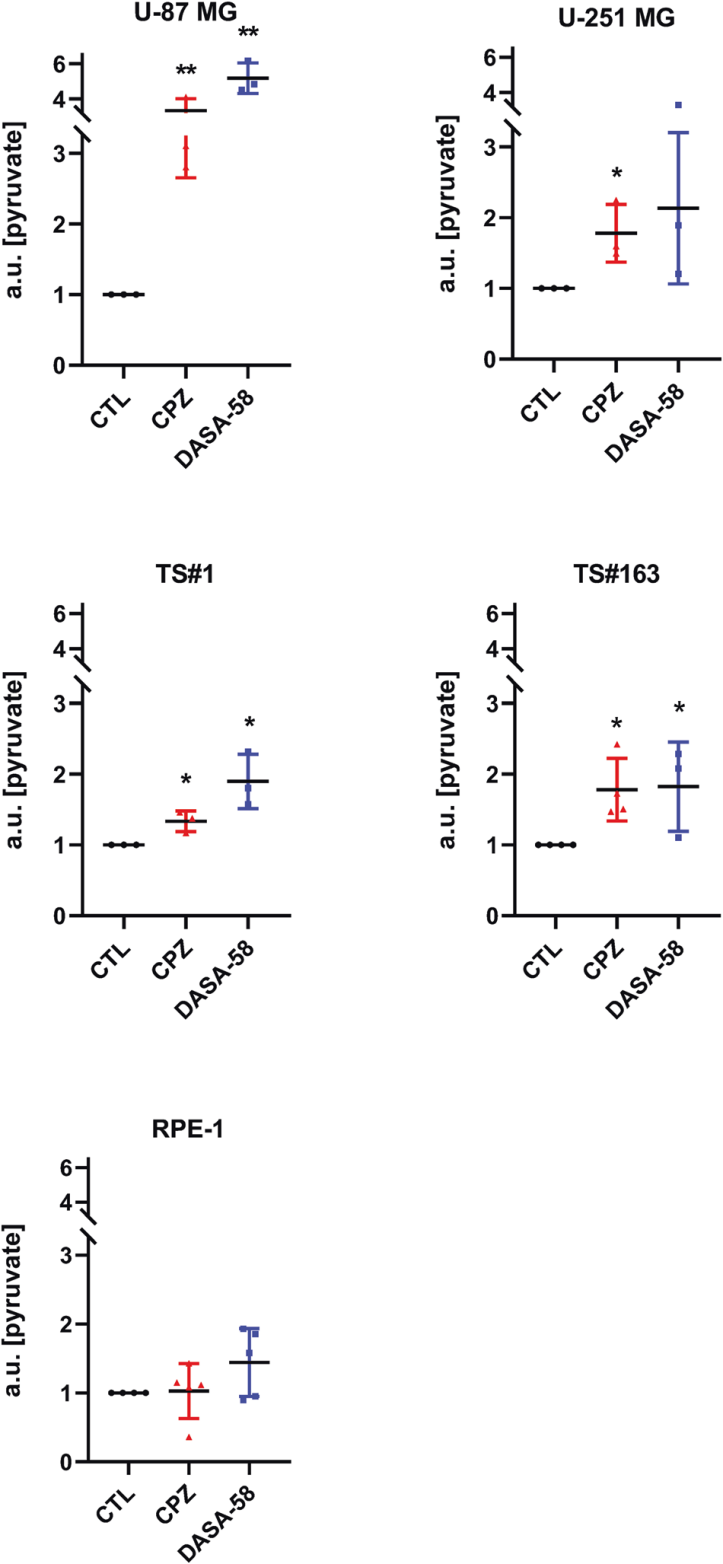
CPZ increases intracellular pyruvate amount in GBM cells. Anchorage-dependent U-87 MG and U-251 MG GBM cells and RPE-1 non-cancer cells were exposed to CPZ or solvent (CTL) for 10 min, while neurospheres TS#1 and TS#163 were exposed for 20 min. As a reference, all cell lines were exposed, under the same conditions, to 30 μM DASA-58, a known PKM2 activator. Histograms show the amount of intracellular pyruvate expressed in arbitrary units (a.u.). Asterisks denote statistical significance (*p < 0.05; **p < 0.01). *N* ≥ 3.

Subsequently, we investigated whether the impairment in GBM lactate production elicited by CPZ was associated with a concomitant variation in the intracellular PK activity. For this purpose, we incubated GBM cells in the presence of CPZ (or vehicle for controls) for 10 min for the U-87 MG, U-251 MG and RPE-1 cells and 20 min for the TS#1 and TS#163 neurospheres. Cells were washed, lysed, and then the intracellular pyruvate amount was determined enzymatically as a readout of the total PK enzymatic activity, comprising also the tetrameric form of PKM2. As a reference, in the same experimental set, cells were incubated with DASA-58, a small molecule known to act as an allosteric activator of PKM2 by inducing its tetramerization and, consequently, its enzyme activity within the glycolytic pathway, thus increasing intracellular pyruvate amount [21]. Exposure to CPZ or DASA-58 significantly increased intracellular pyruvate content in all four GBM cells, whereas no significant variations were observed in RPE-1 non-cancer cells (Fig. 4).

These results evoke an effective role of CPZ in reprogramming glucose catabolism in GBM cells, likely via an allosteric activation (tetramerization) of PKM2. Overall, a decrease in the Warburg effect could be envisaged.

### CPZ decreases nuclear PKM2 amount in GBM cells

**Fig. 5:**
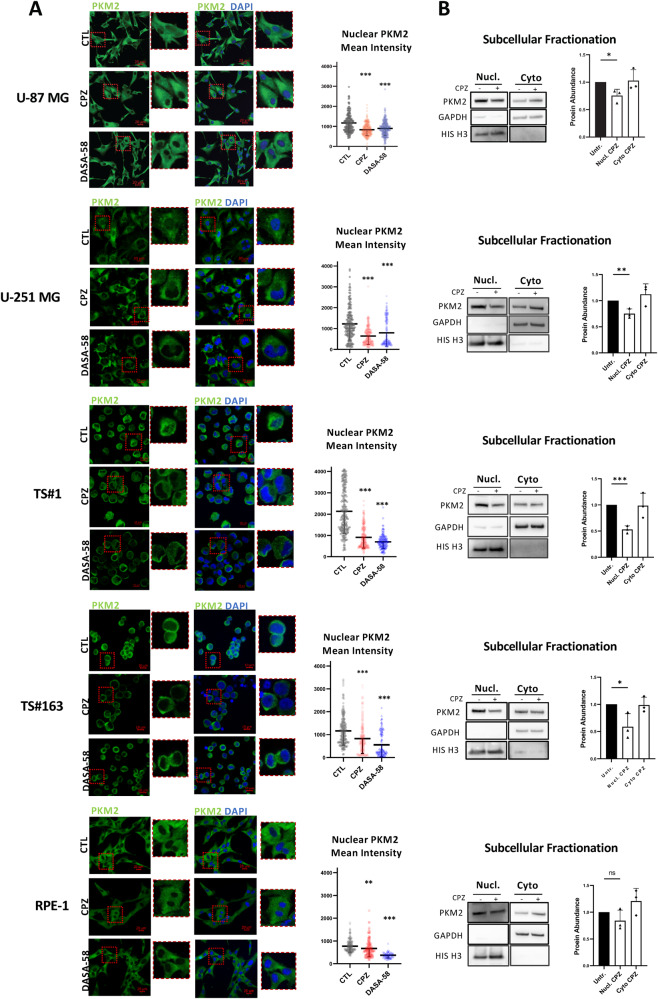
CPZ produces a decrease in nuclear PKM2 concentration in GBM cells. **A** Representative confocal microscopy images of U-87 MG, U-251 MG, TS#1, and TS#163 GBM cell lines and RPE-1 non-cancer cells untreated, CPZ-treated or DASA-58-treated. Green fluorescence shows PKM2, while merging with DAPI (blue) highlights the nuclear structures. The smaller pictures at the right of each image (zoom) reproduce at higher magnification the contents of the red square in each bigger microphotography. Histograms on the right quantify the reduction in nuclear PKM2 mean intensity in CPZ-treated cells (red) or in DASA-58-treated cells (light blue) when compared with controls (black), as evaluated via the microscope software. *N* ≥ 150 nuclei for CTL and CPZ and ≥80 for DASA-58-treated cells. Scale bars are shown in each microphotography. Asterisks denote statistical significance (*p < 0.05; **p < 0.01; ***p < 0.001). **B** The protein levels of PKM2 were assessed using western blotting after subcellular fractionation in all of the cell lines mentioned above. To normalize the results, GAPDH and H3-Histone were used for enriched cytoplasmic and nuclear protein lysates, respectively. Representative western blots on the left show a significant decrease in nuclear PKM2 levels after exposure to CPZ for GBM cells and neurospheres. Histograms on the right quantify the expression levels of PKM2 protein, determined by western blotting, in CPZ-treated GBM cells and in the non-cancer RPE-1 cell line compared to their untreated counterparts (*p < 0.05; **p < 0.01;***p < 0.001). *N*=3.

We then evaluated CPZ-dependent changes in nuclear PKM2 amounts by confocal microscopy. We measured the mean fluorescence intensity of PKM2 signal in the nuclei following 48 h CPZ treatment. Figure 5A shows representative images of anchorage-dependent U-87 MG and U-251 MG GBM cells, TS#1 and TS#163 neurospheres and RPE-1 non-cancer cells, after staining with a fluorescent anti-PKM2 MoAb (green) and with DAPI to highlight nuclei (blue), respectively. For each cell line, PKM2 and merged PKM2 + DAPI staining in control cells and CPZ-treated cells is also shown. As a functional control, cells were also treated with 30 μM DASA-58, which, as expected [21], reduced nuclear PKM2 amount likely by inducing its tetramerization. Histograms represent the average evaluation of PKM2 nuclear content in ≥150 nuclei for CTL and CPZ treated cells and ≥80 for DASA-58-treated cells.

These results were also validated via two alternative methodologies for nuclear PKM2 determination. In the first one, cells were treated with solvent or CPZ as above; then cell fractionation was accomplished, in order to obtain enriched nuclear and cytoplasmic fractions to be further assayed by western blot to obtain a semi-quantitative determination of PKM2. The results showed a marked impoverishment of nuclear PKM2 after exposure to CPZ for all GBM cells and neurospheres, while RPE-1 non-cancer cells displayed a less evident effect of the drug (Fig. 5B). In the second one, all anchorage dependent cells, i.e., U-87 MG, U-251 MG GBM cells and RPE-1 non-cancer cells underwent nuclear isolation, as specified in Materials and Methods, and PKM2 amount was assayed in the nuclear compartment and in the whole cells via flow cytometry. The experimental outcome strengthened the data concerning the ability of CPZ to decrease the content of nuclear PKM2 (Fig. S4).

These results indicate that a significant reduction in nuclear PKM2 was apparent for all the cell lines after treatment with CPZ or DASA-58, albeit less substantial for RPE-1 cells, which in addition displayed an overall lower amount of nuclear PKM2 in untreated cells.

### Effect of CPZ on the functional role of nuclear PKM2

#### CPZ alters the transcriptional pattern downstream of nuclear PKM2

**Fig. 6:**
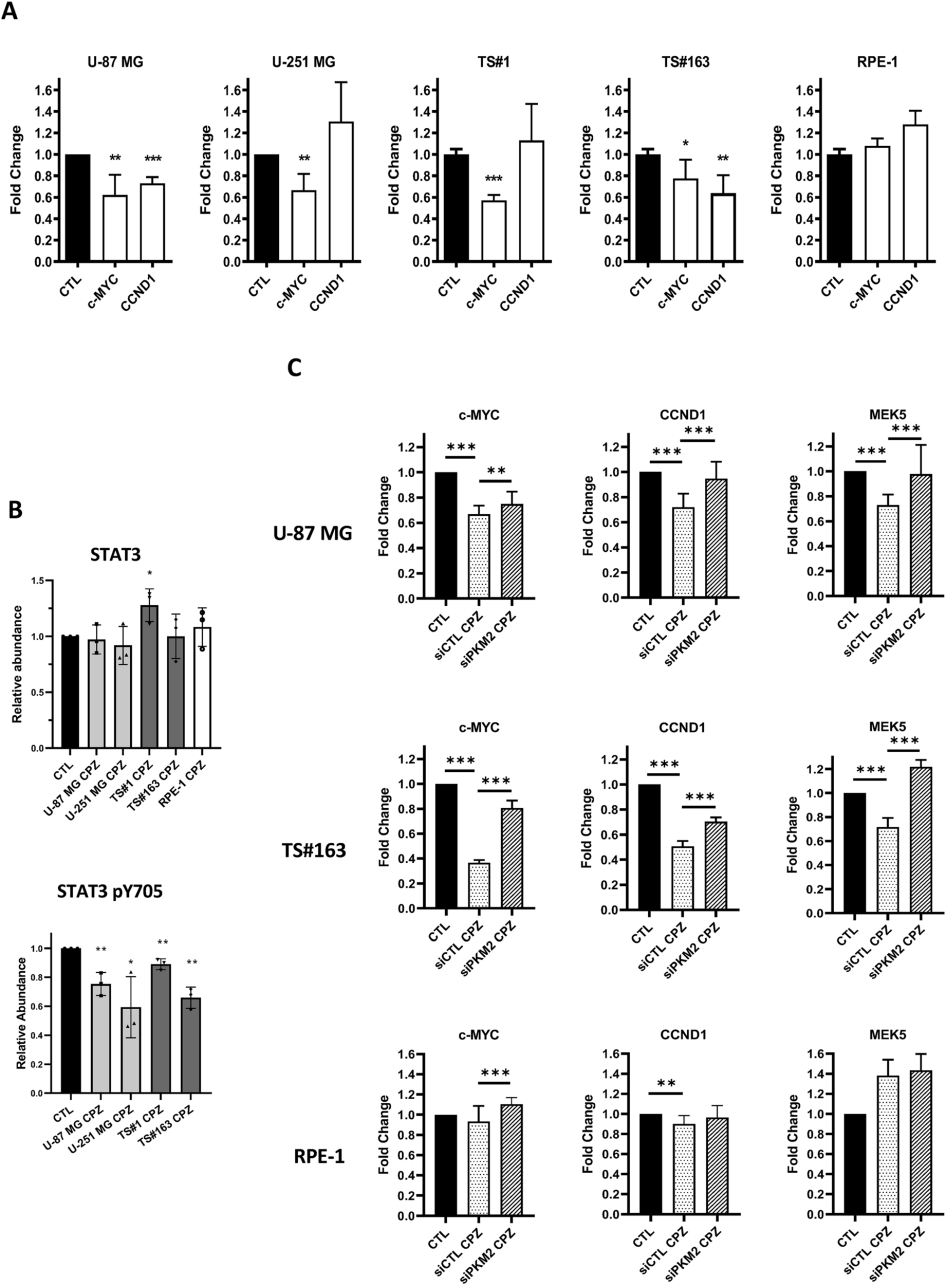
CPZ hinders the function of nuclear PKM2. **A** Expression of *c-MYC* and *CCND1* target genes, as assessed by RT-qPCR, in all the GBM cells and in the RPE-1 cell line. *c-MYC* and *CCND1* expression in untreated cells is normalized to 1.0 (black bars), while their expression under the effect of CPZ are reported as percent variations (white bars). *N* ≥ 10. **B** STAT3 total protein amount and STAT3 pY705 amount as assessed via western blot in all the GBM cells and in the RPE-1 cell line. *N* = 3. **C** U-87 MG, TS#163 GBM cells, and RPE-1 non-cancer cells were treated with siRNA Control or siRNA-PKM2 and exposed to CPZ or solvent for control. Histograms indicate the levels of *CCND1*, *c-MYC*, and *MEK5* mRNA expression, as assessed by RT-qPCR when PKM2 expression was downregulated. The histogram bars related to the control (CTL) values, normalized to 1.0 (solid black), refer to the untreated siRNA Control and PKM2 silenced cells. In all the panels, asterisks denote statistical significance (*p < 0.05; **p < 0.01; ***p < 0.001). *N* ≥ 10.

PKM2 exerts its pro-tumor activity as a dimer, which can acquire nuclear localization, protein kinase activity and associate with various transcription factors [22, 23]. Under the effect of CPZ, PKM2 downstream transcription pattern appeared modified, showing an overall repression of *c-MYC* and *CCND1* transcription, two genes under the control of β-catenin [24], which in turn is a factor activated by nuclear (dimeric) PKM2 [25, 26]. In our setup, *c-MYC* transcription appeared significantly down-regulated in 4/4 (U-87 MG, U-251 MG, TS#1 and TS#163) and *CCND1* in 2/4 (U-87 MG and TS#163) GBM cell lines. These results provide a functional, albeit indirect, link between CPZ and decreased PKM2 nuclear localization. In RPE-1 cells, no significant effects were detectable (Fig. 6A).

#### CPZ influences nuclear PKM2 kinase activity

Dimeric PKM2 is a nuclear protein kinase, where its relevant substrate is the transcription factor STAT3, which is activated by its phosphorylation at Y705 [23], thus promoting *MEK5* transcription and oncogenesis [22, 27]. Although CPZ did not affect total amounts of STAT3 protein in exposed GBM cells, western blot analysis showed a significant decrease in STAT3 pY705 (Fig. 6B).

These results are in line with both decreased amounts of PKM2 in the nuclear compartment and reduced PKM2 protein kinase activity. In the RPE-1 cell line, STAT3 protein, though expressed, appeared undetectable in its phosphorylated form. Representative western blot analyses of STAT3 and STAT3 pY705 in control and CPZ-treated cells are shown in Fig. S5.

### PKM2 is a relevant target of CPZ

To further investigate the interference of CPZ with PKM2 nuclear activity, we silenced PKM2 expression in two GBM cells (U-87 MG and TS#163) and assessed its nuclear activity. As compared with control siRNA, PKM2 silencing resulted in a remarkable reduction of PKM2 protein and mRNA expression in U-87 MG GBM cells, in TS#163 neurospheres and in RPE-1 non-cancer cells, as evaluated via western blotting and RT-PCR, respectively (Fig. S6). Under these conditions, we exposed U-87 MG and TS#163 GBM cells, either siRNA-control or PKM2-silenced, to CPZ and assessed gene expression of *CCND1*, *cMYC*, and also the STAT3 downstream gene *MEK5*. While, in GBM cells, CPZ downregulated the expression of these genes in siRNA control cells, the effect of the drug was significantly lower or missing in siRNA-PKM2 cells. *CCND1*, *cMYC*, and *MEK5* transcription in RPE-1 cells were less influenced by PKM2 silencing (Fig. 6C). In all evaluated cases, the expression fold-changes in PKM2-silenced cells are referred to the corresponding untreated cells.

The clear drop of CPZ-dependent effects in siRNA-PKM2 GBM cells, points again to PKM2 as a major cellular target of CPZ in these cells.

### CPZ binds PKM2 tetramer in the same binding pocket used by other known activators

To identify the PKM2 amino acid residues that interact with the activators in the binding pocket, we analyzed in silico all the experimental structures related to PKM2 tetramer, complexed with activators already reported in PDB, to identify the PKM2 amino acid residues interacting with the activators in the binding pocket. This analysis evidenced that each PKM2 monomer binds a fructose 1,6-bisphosphate (FBP) molecule at an allosteric site located between three amino acid regions (431-437, 482-489, 514-522). In contrast, all the synthetic activators bind to another allosteric site located at the dimer interface of PKM2 and distinct from the FBP binding site (Table S2).

**Fig. 7:**
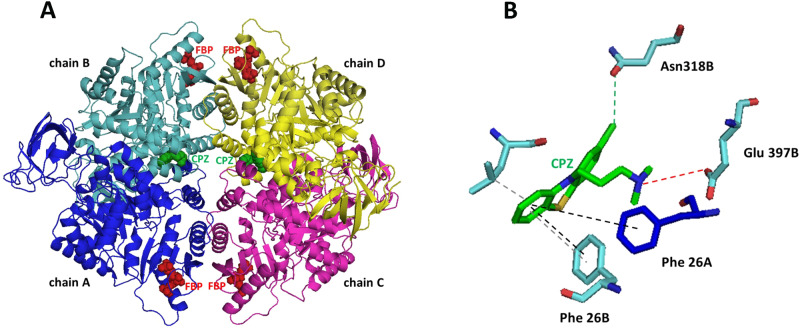
CPZ binds PKM2 tetramer in the same binding pocket of other known activators. **A** Molecular structure of the PKM2/CPZ complex. Chain A is shown in blue, chain B in cyan, chain C in magenta, and chain D in yellow. Four FBP and two CPZ molecules are reported as red and green spheres, respectively. **B** Snapshot of the interaction between chlorpromazine molecule and the chains A and B evidencing stacking and hydrophobic interactions, hydrogen and halogen bonds by dashed black, grey, red, and green lines, respectively.

Molecular docking simulations were performed as reported in Materials and Methods. The more energetically stable structure of the obtained complex showed that two CPZ molecules fit into the binding pocket of the other activators (Fig. 7A). Moreover, the protein-drug interaction appeared stabilized by four hydrophobic interactions, two H-bonds, four π-stacking interactions, and a halogen bond (Fig. 7B).

Indeed, by comparing our CPZ/PKM2 complex with those already reported in PDB for other compounds, we can underline that the affinity energy of our complex falls within the range of values obtained for most complexes and higher only in the case of activators composed by a larger number of atoms and functional groups. Finally, our complex has the maximum number of π-stacking interactions compared with known PDB structures, as the CPZ molecule is a polycyclic aromatic compound containing a linear tricyclic system consisting of two benzene rings joined by a para-thiazine ring (Table S3).

Therefore, these results show a specific interaction between two CPZ molecules and PKM2 and support the ability of the drug to act as a PKM2 allosteric activator.

## Discussion

PKM2 plays a pivotal role in cancer cell bioenergetics, ultimately governing the Warburg effect, i.e., the high glucose consumption and lactate production that most cancer cells display even in the presence of adequate oxygen concentrations and intact cellular machinery devoted to mitochondrial ATP production [17, 25, 28, 29]. Warburg effect is also essential for protecting cancer stem cells from their elevated ROS production [30].

Such a peculiar bioenergetics asset displayed by most cancer cells, including GBM, could represent a vulnerability when a compound is able to affect their energy metabolism.

Our results highlight the ability of CPZ to interfere with GBM energy metabolism and key signal transduction pathways involved in the anabolic processes used by these cells to facilitate the synthesis of building blocks for biomass generation.

Collectively, these results suggest the potential anticancer role of CPZ and highlight PKM2 as a novel target for this medication. The overall picture of the CPZ-induced modifications indicates the ability of this drug to slow down cellular anabolism, attributable to a possible energy shortage (see below) and to elicit a stress response in GBM cells, which stimulate an autophagic pathway in search for rescue strategies, in line with previous findings [13, 31].

In particular, the effects of CPZ in impairing the PI3K/mTOR pathway, activating autophagy, and reducing lactate extracellular release seem attributable to the interaction we recognized between the drug and PKM2. Of note, the CPZ-induced decrease in extracellular lactate release could radically modify the peritumoral environment and make it less fit for cancer growth and progression [32].

Noticeably, CPZ appeared less toxic for the RPE-1 non-cancer cells. A reason to explain the selective toxicity of CPZ toward GBM cells could be envisaged in the peculiar pattern of expression of PK isoforms postulated for cancer cells, where a higher PKM2/PKM1 ratio is widely described [33, 34]. Indeed, PKM2 tetrameric activators operate to favor the generation of a tetramer from two PKM2 dimers and have no described effect on PKM1 or other PK isoforms constitutively present as a tetramer. Therefore, these drugs do not modify PK activity sustained by PKM1 or other isoforms in cells expressing negligible or null amounts of PKM2 [33, 34], allowing us to assume that PKM1 could safeguard non-cancer cells. Conversely, PKM2 tetramerization can hinder major malignant features in GBM [33].

The results reported here show the multiple pharmacodynamic activities of CPZ in hindering the survival ability of GBM cells, while displaying less toxicity toward the RPE-1 non-cancer neuroectodermal cells.

Although we hypothesize that PKM2 tetrameric activation can reverse the Warburg effect and decrease lactate production, we cannot rule out other possible effects of CPZ that could contribute to decreased extracellular lactate concentration, such as LDH, MCT1/4, and/or GLUT inhibition or interference with the other glycolytic regulatory enzymes (HK and PFK). We are currently investigating the potential effects of CPZ on these other metabolic regulators and metabolites using ^13^C NMR spectroscopy.

In our setup, CPZ behaved very similarly to the PKM2 allosteric activator DASA-58, a compound known to favor the tetrameric form of PKM2 and consequently reduce its nuclear content and the Warburg effect. DASA-58 impedes cancer cell growth in preclinical in vitro and in vivo models [33]; however, we are unaware of clinical trials involving this molecule or other known tetrameric PKM2 activators in GBM therapy. In converse, CPZ can claim its status of an old and thus repositionable drug, being a medication widely used mainly for the therapy of neuropsychiatric disorders, making this compound eligible for immediate use in clinical experimentation.

We should also consider that no related toxicity has been described in psychiatric patients undergoing long-term therapy with CPZ at high doses. Incidentally, there are anecdotal reports of a reduced cancer rate in patients under therapy with CPZ [35] and a better GBM clinical course in psychiatric patients on neuroleptics [36].

On these bases, we initiated a Phase II clinical trial, approved by our Institutional Ethics Committee (Comitato Etico Centrale IRCCS - Sezione IFO-Fondazione Bietti, Rome, Italy) on September 6, 2019 (EudraCT # 2019-001988-75; ClinicalTrials.gov Identifier: NCT04224441). In this trial, CPZ has been added to the standard GBM treatment in patients carrying a tumor with a hypo-methylated *MGMT* gene promoter and thus characterized by resistance to TMZ and poorer prognosis [37]. The results of this trial will be available at the end of 2023. Independently, another Phase I clinical trial (ClinicalTrials.gov Identifier: NCT05190315) is currently investigating the use of CPZ throughout the standard of care for GBM therapy.

Since the whole process required to develop and bring new drugs to clinics is currently extremely long and expensive [38, 39], experimental and clinical investigators are strongly motivated to consider drug repositioning/repurposing [40], especially now that novel bioinformatics and multi-omics platforms can help unveil the potential of several well-known medications. This approach may also provide further benefits, including safety, a faster track to clinical use and a relative inexpensiveness, with the aim to provide novel and effective therapeutic approaches for GBM patients.

## Materials and methods

### Cell lines

Anchorage-dependent GBM cell lines U-87 MG and U-251 MG, anchorage-independent TS#1 and TS#163 neurospheres, and anchorage-dependent hTERT-immortalized human retinal pigment epithelial cells hTERT RPE-1 (henceforth RPE-1) are described and cultured as previously reported [13].

### Drugs

CPZ was purchased as “Largactil” from Teofarma S.R.L., Valle Salimbene (PV), Italy, as a 25 mg/ml solution (78 mM). CPZ doses used throughout the paper refer to the IC30 for each cell line, as determined by their 48-h exposure to the drug (see Table S1, where IC50 doses are also reported). DASA-58 was purchased as a powder from Selleckchem, Houston, TX, USA, and dissolved in DMSO as a 50 mM stock solution.

### RPPA

Reverse-Phase Protein microArrays (RPPA) analysis was performed based on established protocols. Anchorage-dependent GBM cells U-87 MG, U-251 MG, and TS#1 and TS#163 neurospheres were seeded onto 6-well microtiter plates (3.5 × 10^3^ cells/well) and treated either with vehicle or CPZ at the cell line-specific IC30 and IC50 for 2 and 8 h and processed as described [41–43]. Cells from three different passages were used for biological replicates of individual experimental conditions. After cell lysis and protein determination, printing was performed using an Aushon 2470 arrayer equipped with 185 μm pins (Aushon Biosystems, Billerica, MA) and samples were immobilized onto nitrocellulose-coated glass slides (GRACE Bio-Labs, Bend, OR) in triplicate spots along with a set of reference standard lysates, i.e. 10-point dilution curves of HeLa + Pervanadate (BD, Franklin Lakes, NJ), Jurkat + Etoposide (Cell Signaling, Danvers, MA), Jurkat + Calyculin A (Cell Signaling), A431 + Pervanadate (Santa Cruz Biotechnologies) and A431 + EGF (BD, Franklin Lakes, NJ). Total protein content of printed slides was evaluated by Sypro Ruby Protein Blot Stain (Invitrogen, Carlsbad, CA). Printed slides were then subjected to unmasking, then immunostaining was carried out by incubation with primary antibody and subsequently biotinylated goat anti-rabbit IgG H + L (1:7500) (Vector Laboratories, Burlingame, CA) or rabbit anti-mouse IgG (1:10) (DAKO). Signal amplification was performed by DAKO GenPoint kit (Agilent, CA, USA) followed by incubation with a fluorescently labelled tertiary reagent, i.e., streptavidin-conjugated IRDye680LT (LI-COR Biosciences, Lincoln, NE). Negative control slides were incubated with secondary antibody alone. Total protein and immunostained slides were imaged by a Tecan power scanner™ (Tecan Group Ltd, Switzerland) and digital 16 bit images were analyzed by MicroVigene v5.2 (VigeneTech, Carlisle, MA) software for spot detection, local background subtraction, negative control subtraction, replicate averaging and total protein normalization. RPPA data resulting from image analysis and processing are referred to as normalized RPPA intensity or levels and are expressed in arbitrary units (A.U.). RPPA data analysis and graphical representation was performed by means of ‘R’ v4.1.2 (https://www.r-project.org/) (R Foundation for Statistical Computing) and ‘RStudio’ v2022.07.1 https://www.rstudio.com/ (RStudio) using the following packages: base, tidyverse, RColorBrewer, ggnewscale and trelliscopejs.

### Identification of potential CPZ protein targets

To identify potential cellular targets of CPZ, we employed ABPP in competitive mode. Multiple aliquots of the same native GBM cell lysates were incubated with increasing concentrations of CPZ (5-40 µM) and then mixed with an ATP-mimicking insoluble probe. Proteins whose ATP-binding ability was influenced by the drug were picked and identified by MALDI-MS and MS/MS analysis. All these procedures were performed as described [44].

### Evaluation of metabolic parameters

#### Glycolytic proton efflux rate (glycoPER) and oxygen consumption rate (OCR)

The most recent procedure to calculate glycolytic activity is the glycolytic proton efflux rate (glycoPER), which improves the measurement of extracellular lactate release by accounting for buffering and measuring and subtracting CO_2_‑dependent acidification (https://www.agilent.com/cs/library/whitepaper/public/whitepaper-improve-quantification-of-cellular-glycolytic-rate-cell-analysis-5991-7894en-agilent.pdf).

GlycoPER and OCR measurements were performed using the Seahorse XFp Real-Time Cell metabolic analyzer (Agilent Technologies, Santa Clara, CA 95051, USA) following manufacturer’s instructions (Supplementary Materials and Methods). The output generated by the Agilent Seahorse Analytics web-based software has been further processed by means of the Prism v9 (GraphPad Software Inc., San Diego, CA).

#### Intracellular Pyruvate determination

The amount of intracellular pyruvate was determined using the enzymatic Pyruvate Kinase Activity Assay MAK072 from Sigma-Aldrich Merck KGaA, Darmstadt, Germany, following manufacturer’s instructions (Supplementary Materials and Methods).

### RNA extraction and RT-PCR

U-87 MG and U-251 MG anchorage-dependent cells and TS#1 and TS#163 neurospheres were treated with CPZ for 24 h, while control cells were treated with the same volume of vehicle. Total RNA was extracted using miRNeasy Extraction Kit (QIAGEN, Hilden, Germany), and RNA concentration was determined. After reverse RNA transcription, real-time (RT) polymerase chain reaction (PCR) analyses were performed to determine nuclear PKM2 downstream transcriptional activity. All RT-PCR data were quantified using the 2^−ΔΔCT^ method, and CT values were normalized to GAPDH. Values represent fold changes related to control cells, arbitrarily reported as 1.0.

Primers are described in Supplementary Materials and Methods.

### Confocal microscopy

The procedures implemented for confocal microscopy are described in Supplementary Materials and Methods.

### Western blotting

Cells were lysed in RIPA, separated by means of a 4–12% precast polyacrylamide gel and transferred onto a PVDF membrane, which was probed using the following rabbit MoAbs: anti-STAT3 (Cell Signaling Technology, Danvers, MA, 1:1000); anti-STAT3 pY705 (Cell Signaling Technology, 1:1000); anti-PKM2 (Cell Signaling Technology, 1:1000); anti-histone H3 (Cell Signaling Technology, 1:1000) and the mouse MoAbs anti-GAPDH (Sigma-Aldrich, St. Louis, MO, 1:10 000) and anti-β-actin (MP Biomedicals, Irvine, CA, 1:10 000).

### Nucleus/cytoplasm cell fractionation

Nucleus and cytoplasm cell fractionation was obtained using NE-PER Nuclear and Cytoplasmic Extraction Kit (Thermo Fisher Scientific) following manufacturer’s instructions. Recovered protein fractions were processed for western blot as described above. Nuclear PKM2 signals were normalized against Histone H3, while cytosolic ones via GAPDH.

### Cytofluorimetric PKM2 determination in isolated nuclei

Control and CPZ-treated cells were washed twice in PBS, harvested by a policeman and collected by centrifugation. Cells were then resuspended in homo-buffer [10 mM] Hepes, pH 7.4; 1 mM EGTA, 0.1 M sucrose, 5% BSA, 1 mM PMSF and a complete protease inhibitor cocktail (Roche, Basel, Switzerland) and maintained on ice for 10 min. Next, cells were homogenized with 70 strokes of a Teflon homogenizer with B-type pestle on ice to break intact cells and obtain isolated nuclei. During this phase, cells were checked via Trypan blue staining until at least 90% of isolated nuclei were obtained. Nuclei were collected by centrifugation at 10,000 ×g at 4 °C for 10 min and pellet was fixed and permeabilized with 2% paraformaldehyde for 30 min and permeabilized by 0.5% (vol/vol) Triton X-100. After washings in PBS, cells were stained with a rabbit anti-PKM2 MoAb (Cell Signaling Technology, 1:100), a mouse anti-Lamin A/C (Santa Cruz Biotechnology, Dallas, TX, 1:100) as a positive control for nuclei, followed by anti-mouse-PE and anti-rabbit-Cy5. After washing, cells were labelled with a monoclonal anti-Bcl2 FITC-conjugated antibody (Dako, Agilent, Santa Clara, CA) as a cytosolic marker. As negative controls, we used cells incubated with specific isotype followed by secondary antibodies, and with the isotype-matched FITC-conjugated monoclonal antibody. After labeling, samples were washed and immediately analyzed on a cytometer.

### siRNA transfection

Anchorage-dependent cells were seeded in 35-mm diameter dishes; on the following day, cells were transfected with 10 nM siRNA-PKM2 (siPKM2) or negative control siRNA using Lipofectamine RNAiMAX (Invitrogen Thermo Fisher Scientific). Small interfering-PKM2 (PKM2 Silencer Select Validated siRNA) and negative control siRNA (Silencer Select Negative Control #1 siRNA) were purchased from Ambion (Austin, TX, USA). After 48-h transfection, cells were treated with CPZ or vehicle for 24 h, collected, and used for RT-PCR and/or western blot analysis. When cells were employed for Seahorse determinations, cells were reseeded in the proprietary miniplates, and measurements were carried out after additional 24 h.

Neurospheres were plated in Stem Medium containing 3% Matrigel (Corning Matrigel Growth Factor Reduced Basement Membrane Matrix, Merck, Darmstadt, Germany); the subsequent day, cells underwent PKM2 silencing as described above for anchorage-dependent cells.

### Molecular docking simulations

To predict CPZ’s best binding to the tetrameric PKM2 structure, molecular docking studies were performed using AutoDock 4.2.6 tool [45] and employing the crystal structure of the PKM2 tetramer (PDB code: 5X1W) as the target [46] and CPZ structure retrieved from ZINC20 database as the ligand [https://pubs.acs.org/doi/10.1021/acs.jcim.0c00675]. The docking protocol was performed by extracting a co‐crystallized ligand and docking two CPZ molecules into the active pocket in each PKM2 dimer. See Supplementary Materials and Methods for further details.

The 10 best poses of each CPZ molecule were clustered using an RMSD value <2.0 Å. The best-docked conformation of two CPZ molecules on two dimers of PKM2 was selected in the obtained cluster based on the binding affinity (expressed in kcal/mol) and the number of ligand–protein interaction residues, H-bonds, salt bridges, and hydrophobic and stacking interactions by AutoDock4.2.6 [45], LIGPLOT [47], PLIP [48] and PRODIGY [49].

### Statistical analysis

Three independent experiments were carried out for confocal microscopy images, and statistical analysis was performed, on ≥150 nuclei for CTL and CPZ and ≥80 for DASA-58-treated cells, using an unpaired Student’s *t*-test (Prism v9).

Pyruvate concentrations and transcription levels expression determinations are described as mean ± Standard Deviation (SD). Up- or down-regulations relative to controls were analyzed using the Student’s two-tailed *t*-test (Prism v9). Asterisks denote statistical significance (*p < 0.05; **p < 0.01; ***p < 0.001).

### Reporting summary

Further information on research design is available in the Nature Research Reporting Summary linked to this article.

### Supplementary information

Supplemental Material

Supplemental Figures

Original Data File

Reporting Summary

## Data Availability

Raw data are available at the following link: https://gbox.garr.it/garrbox/s/WVF3cy2xWKocZvi.
