# Supplementary material for: Chlorpromazine affects glioblastoma bioenergetics by interfering with pyruvate kinase M2
Source: Cell Death Dis. 2023 Dec 13;14(12):821. doi: 10.1038/s41419-023-06353-3 (PMC10719363; doi:10.1038/s41419-023-06353-3)
Supplement: Supplementary file 1 — Supplemental Material [file 41419_2023_6353_MOESM1_ESM.docx]

**Supplementary Tables**

**Table S1**

CPZ IC30 and IC50 values for each of the cell lines utilized. They represent the drug concentrations that inhibit the cell viability by 30% and 50% after 48 h of exposure.

| ***Cell Line*** | ***CPZ IC30 (μM)*** | ***CPZ IC50 (μM)*** |  |
| --- | --- | --- | --- |
| **U-87 MG** | 7.4 ± 1.2 | 10.4 ± 1.3 | **Anchorage-dependent cells** |
| **U-251 MG** | 7.0 ± 1.1 | 10.6 ± 1.2 |  |
| **RPE-1** | 10.4 ± 1.1 | 13.7 ± 1.6 |  |
| **TS#1** | 14.8 ± 1.0 | 21.6 ±1.1 | **Neurospheres** |
| **TS#163** | 10.9 ± 1.2 | 15.4 ± 1.4 |  |

**Table S2**

Amino acids of the PKM2 dimer composed of A and B chains at the interface with several activators in the known PDB structures and in the PKM2/CPZ complex by the LigPlot tool.

|  | **3GQY** | **3GR4** | **3H6O** | **3U2Z** | **3ME3** | **6TTQ** | **5X1W** | **4JPG** | **4G1N** | **PKM2/CPZ complex** |
| --- | --- | --- | --- | --- | --- | --- | --- | --- | --- | --- |
| **Phe26 A** | x | x | x | x | x | X | x | x | x | x |
| **Leu27 A** | x | x |  | x |  |  | x |  |  | x |
| **His29A** |  |  |  |  |  |  |  | x |  |  |
| **Met30 A** | x |  |  | x |  |  |  |  | x | x |
| **Lys311 A** |  |  |  |  |  |  | x |  |  | x |
| **Asn318 A** | x |  |  |  | x |  |  |  |  |  |
| **Leu353 A** |  | x | x |  | x |  |  | x |  |  |
| **Asn350 A** |  |  |  |  |  |  | x |  |  |  |
| **Leu353 A** |  |  |  |  |  |  | x |  | x |  |
| **Asp354 A** | x |  |  |  | x |  | x |  |  |  |
| **Ala388 A** |  |  |  |  |  | x | x | x |  |  |
| **Ile389 A** |  |  |  |  |  | x | x |  | x | x |
| **Tyr390 A** | x | x | x | x | x | x | x |  | x | x |
| **Gln393 A** | x | x | x | x | x | x | x |  | x | x |
| **Leu394 A** | x | x | x | x | x |  | x |  | x | x |
| **Glu397 A** | x | x | x |  | x |  | x |  | x | x |
| **Phe26 B** | x | x | x | x | x |  | x | x | x | x |
| **Leu27 B** | x |  | x |  | x |  | x |  |  | x |
| **Met30 B** | x |  |  |  | x |  |  |  | x | x |
| **Lys311 B** |  |  |  |  |  | x | x | x | x |  |
| **Asn318 B** | x | x |  |  |  |  |  |  |  | x |
| **Asn350 B** |  |  |  |  |  |  | x |  |  |  |
| **Leu353 B** | x | x |  |  | x |  | x |  | x | x |
| **Asp354 B** | x | x |  | x | x |  | x |  | x | x |
| **Tyr390 B** | x | x | x | x | x |  | x |  | x | x |
| **Gln393 B** | x | x | x | x | x |  | x | x |  | x |
| **Leu394 B** | x | x | x | x | x |  | x |  | x | x |
| **Glu397 B** | x | x |  | x | x |  | x |  |  | x |

**Table S3**

The number of amino acids of the PKM2 involved in π-stacking interactions and H-bonds, and values of affinity energy (expressed in Kcal/mol) between PKM2 and activators in the known PDB structures and PKM2/CPZ complex.

|  | **π-stacking interactions** | **H-bonds** | **Affinity energy (Kcal/mol)** |
| --- | --- | --- | --- |
| **PKM2/CPZ complex** | 4 | 2 | -9.3 |
| **3GQY** | 4 |  | -10 |
| **3GR4** | 3 | 1 | -10.2 |
| **3H6O** | 4 |  | -9.3 |
| **3U2Z** | 4 | 2 | -8.6 |
| **3ME3** | 3 | 2 | -9.5 |
| **6TTQ** |  | 2 | -8.6 |
| **5X1W** | 3 | 4 | -12.4 |
| **4JPG** | 4 |  | -8.3 |
| **4G1N** | 2 | 3 | -9.7 |

**Supplementary Materials and Methods**

**Evaluation of metabolic parameters**

*A. Oxygen consumption rate (OCR) and extracellular acidification rate (ECAR)*

Cells were seeded at 5,000/well (XFp plate) and incubated for 24 h in a humidified 37° C incubator with 5% CO_2_ (DMEM). Prior to performing an assay, the growth medium in the wells was changed with the appropriate assay medium at pH 7.4. All experiments were performed at 37° C.

Each measurement cycle consisted of a mixing time of 3 min and a data acquisition period of about 6 min (21 data points). OCR and ECAR data points refer to the average rates during the measurement cycles. All compounds were added sequentially as follows: CPZ (or medium for the control) was added to the first injection port A (acute injection), rotenone + Antimycin A (AA) were added in the port B, and 2-deoxy-D-glucose (2-DG) was added in the port C.

Based on the time points of rotenone + Antimycin A and 2-DG injection, the basic conditions of glycolysis, maximal glycolytic capacity, and non-glycolytic activity were detected. Then cells were counted to normalize either OCR or ECAR. OCR and ECAR were reported as absolute rates (pmoles/min for OCR and mpH/min for ECAR). Unless otherwise specified, the third measurement of baseline or after the addition of each substrate or compound was used to generate absolute OCR or ECAR values^1^. Each datum was determined at least in triplicate.

*B. Intracellular Pyruvate determination*

Cells were plated in 35-mm ø dishes and treated with drug vehicle alone, CPZ or 30 μM DASA-58 for a time corresponding to 10 min for all anchorage-dependent cells and 20 min for neurospheres. Afterward, treated cells were swiftly pelleted, resuspended in lysis buffer, and plated in a clear 96-well plate for colorimetric assay. Pyruvate concentration was determined by a coupled enzyme assay, resulting in a colorimetric product (570 nm) proportional to the pyruvate amount. Pyruvate concentration was evaluated by interpolating each sample absorbance with a pyruvate standard curve; values were expressed as arbitrary units (a.u.), and results were plotted as histograms.

***RNA extraction and RT-PCR***

Primer pairs used for quantitative real-time PCR:

***CCND1***: fwd-GCGAGGAACAGAAGTGC; rev-GAGTTGTCGGTGTAGATGC

***c-MYC***: fwd-CCACCTCCAGCTTGTACCTG; rev-GAGCAGAGAATCCGAGGACG

***MEK5***: fwd-CCAGAACATGTCCTTGGAAGA; rev-CACCAGCTGAGTGCTAACTCC

***PKM2***: fwd-CTATCCTCTGGAGGCTGTGC; rev-GTGGGGTCGCTGGTAATG

***GAPDH***: fwd-TGACATCAAGAAGGTGA; rev-TCCACCACCCTGTTGCTGTA

***Confocal microscopy***

Cells were fixed in 4% paraformaldehyde for 10 min and then permeabilized in PBS plus 0.1% Triton X-100 for 10 min. Neurospheres were gently cytocentrifuged on a polylysine-coated slide. After blocking with 5% bovine serum in PBS for 1 h, cells were incubated with the primary anti-PKM2 antibody (D78A4, Cell Signaling, Danvers, MA, USA) at a 1:150 dilution at RT for 90 min. Cells were then incubated with Alexa Fluor-488 labeled goat anti-rabbit secondary antibody (A11034, Thermo Fisher) (1:500) at RT. Before imaging, cell nuclei were stained with DAPI.

Immunofluorescence was analyzed using an LSM 880 with an Airy-Scan confocal laser scanning microscope (Carl Zeiss AG, Oberkochen, Germany) equipped with 40× or 63×/1.23 NA oil immersion objectives. The 405 and 488 nm lasers were used to excite the fluorophores.

Fluorescence images of neurospheres were obtained from 20 sequential Z-stack scans in two channels (DAPI, Alexa Fluor-488) and compiled into single images. In contrast, anchorage-dependent cells were scanned only once. Zeiss Zen software (Carl Zeiss, Germany) was used for image acquisition, and Zen 3.6 (Zen Desk) for image analysis. The software automatically drew the Region Of Interest (ROIs) around nuclei; then, PKM2 mean intensity values within the nuclei were exported. Three independent experiments for CPZ and two for DASA-58 were performed for each cell line.

**Molecular docking simulations on PKM2 tetramer and CPZ**

To define the binding pocket of activators in PKM2 structure, we analyzed using the LigPlot tool^2^ all nine known experimental structures related to PKM2 tetramer complexed with activators (PDB code: 3GQY, 3GR4, 3H6O, 3U2Z, 3ME3, 6TTQ, 5X1W, 4JPG and 4G1N) to identify the interacting residues of PKM2 at the interface with the different compounds.

Both PKM2 and CPZ structures were prepared in AutoDock Tools 1.5.6^3^. To CPZ molecules, non-polar hydrogens atoms and Gasteiger charges were added. Polar hydrogen atoms were added to the PKM2 structure, and Kollman and Gasteiger charges were assigned. A grid map in each dimer with a spacing of 0.375 Å and dimensions of 9.28 × 2.53 × 13.43 points, focused on the residues belonging to the binding site of PKM2, as described above, was used to set up the simulations of the binding of CPZ molecules at two binding sites located between the chains A and B in the first dimer and chains C and D in the second dimer.

AutoDock Lamarckian genetic algorithm was performed for conformational search, and the number of docking interactions (defined as run) was set to 100 using a number of generations: 27000; a number of energy evaluations: 2500000; and population size: 150).

**References:**

**1.** Jady AG, Nagy AM, Kohidi T, Ferenczi S, Tretter L, Madarasz E. Differentiation-Dependent Energy Production and Metabolite Utilization: A Comparative Study on Neural Stem Cells, Neurons, and Astrocytes. *Stem Cells Dev.* 2016; 25(13):995-1005.

**2.** Wallace AC, Laskowski RA, Thornton JM. LIGPLOT: a program to generate schematic diagrams of protein-ligand interactions. *Protein Eng.* 1995; 8(2):127-134.

**3.** Forli S, Huey R, Pique ME, Sanner MF, Goodsell DS, Olson AJ. Computational protein-ligand docking and virtual drug screening with the AutoDock suite. *Nat Protoc.* 2016; 11(5):905-919.
