# Supplementary material for: Chlorpromazine affects glioblastoma bioenergetics by interfering with pyruvate kinase M2
Source: Cell Death Dis. 2023 Dec 13;14(12):821. doi: 10.1038/s41419-023-06353-3 (PMC10719363; doi:10.1038/s41419-023-06353-3)
Supplement: Supplementary file 2 — Supplemental Figures [file 41419_2023_6353_MOESM2_ESM.pdf]

Figure S1

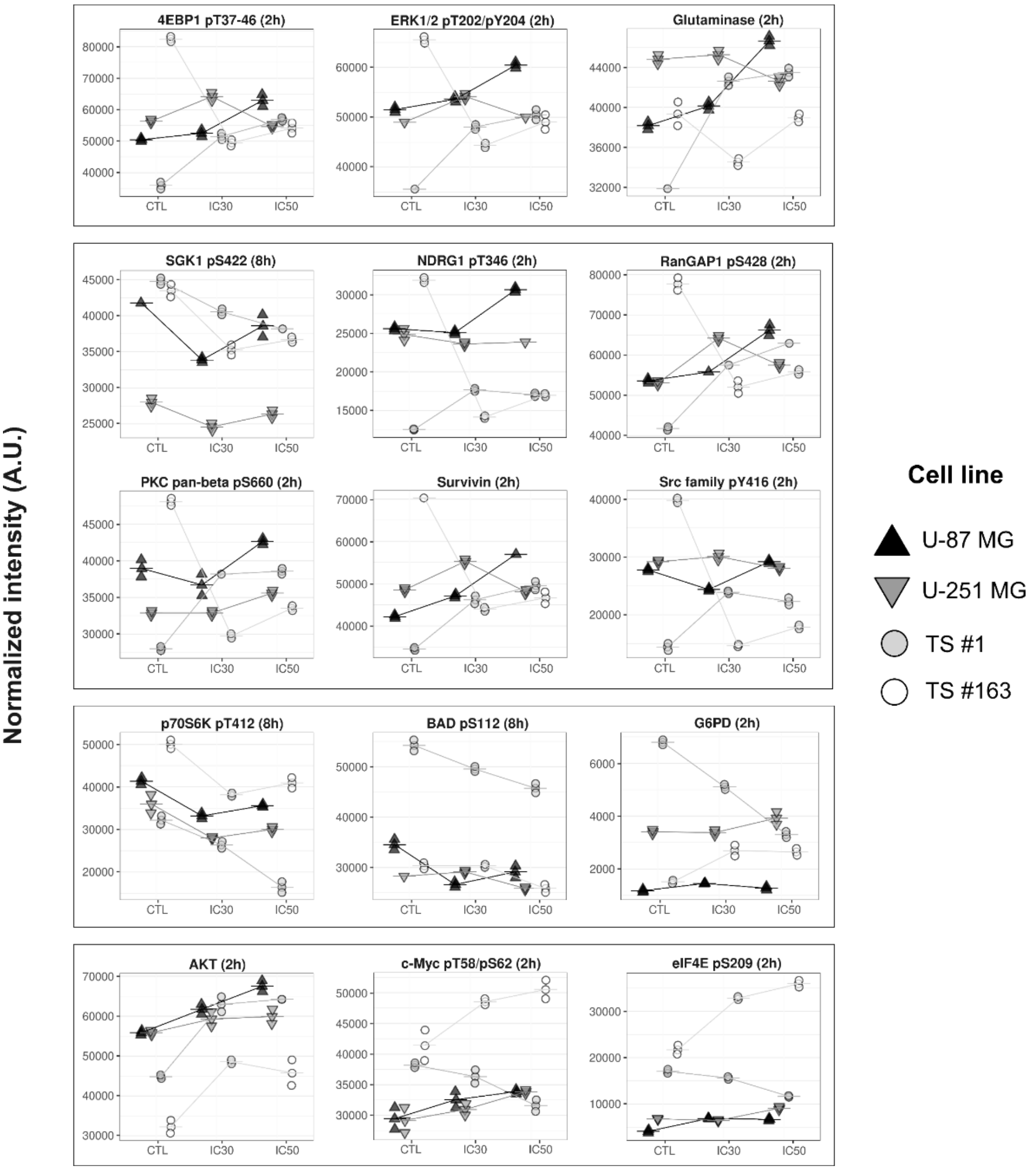

RPPA analysis of anchorage-dependent GBM cells and neurospheres challenged with CPZ. The panels include selected plots of normalized RPPA levels (Arbitrary Units, AU) for endpoints showing patterns of co-regulation in a cell- and time-dependent manner. The determinants shown are involved in key metabolic pathways not discussed in the main text, but may provide support to the experimental data. Measurements are carried over a three-point dose response of CPZ (Control, IC30 and IC50, from left to right) at either 2 or 8 h. N=3.

Figure S2

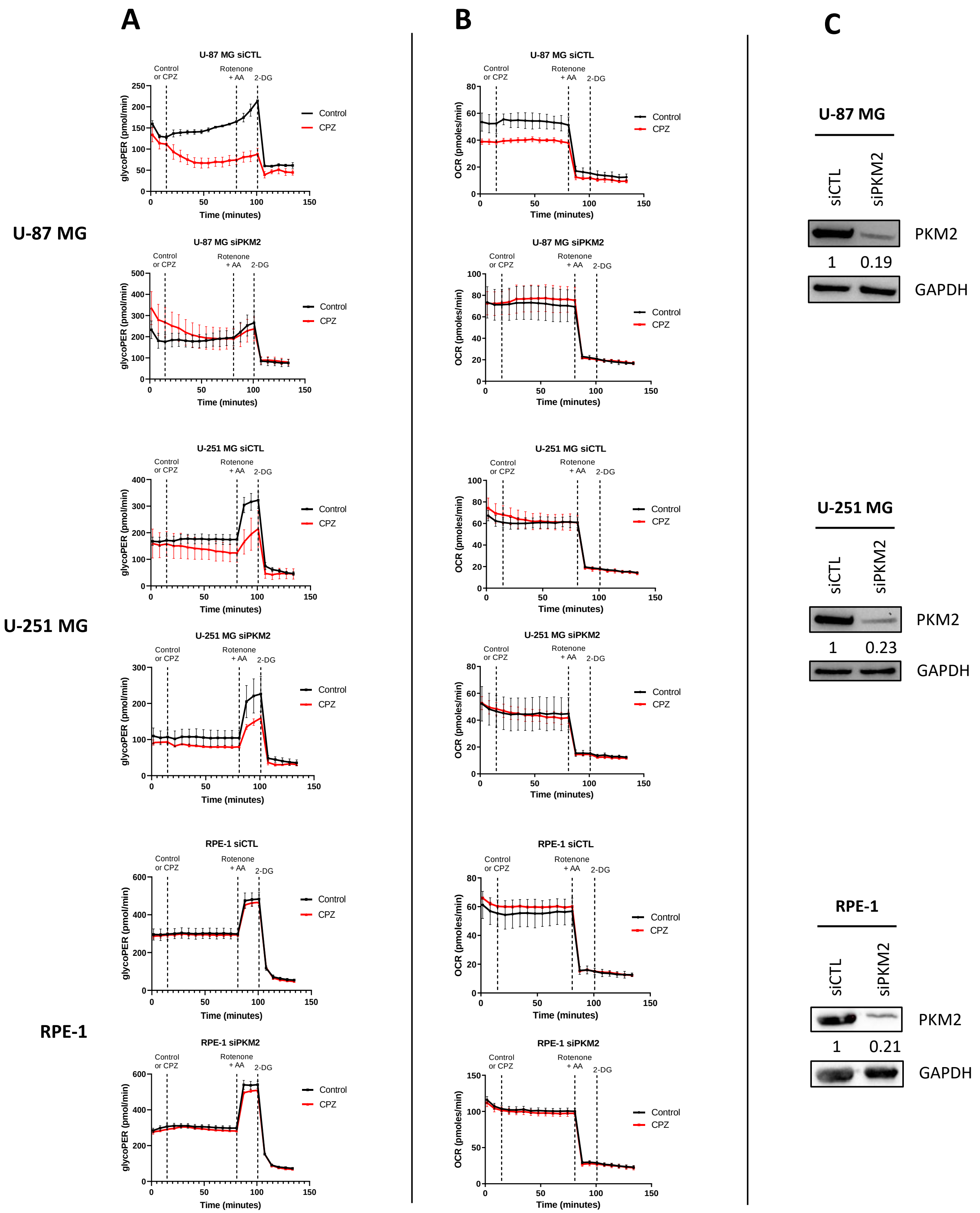

PKM2 is a target of CPZ in hindering glucose metabolism. After PKM2 silencing, cells were analyzed via the Seahorse XFp platform. Red lines represent CPZ-treated cells and black lines control cells. A. GlycoPER plots related to U-87 MG, U-251 MG GBM cell lines and RPE-1 non-cancer cells. B. OCR plots related to U-87 MG, U-251 MG GBM cell lines and RPE-1 non-cancer cells. All experiments were performed twice in triplicate. Representative graphs are shown here; dots and vertical bars indicate mean ± SD. Raw data from all experiments are available in Supplementary Material. C. Representative western blots showing PKM2 silencing in the corresponding experiments.

Figure S3

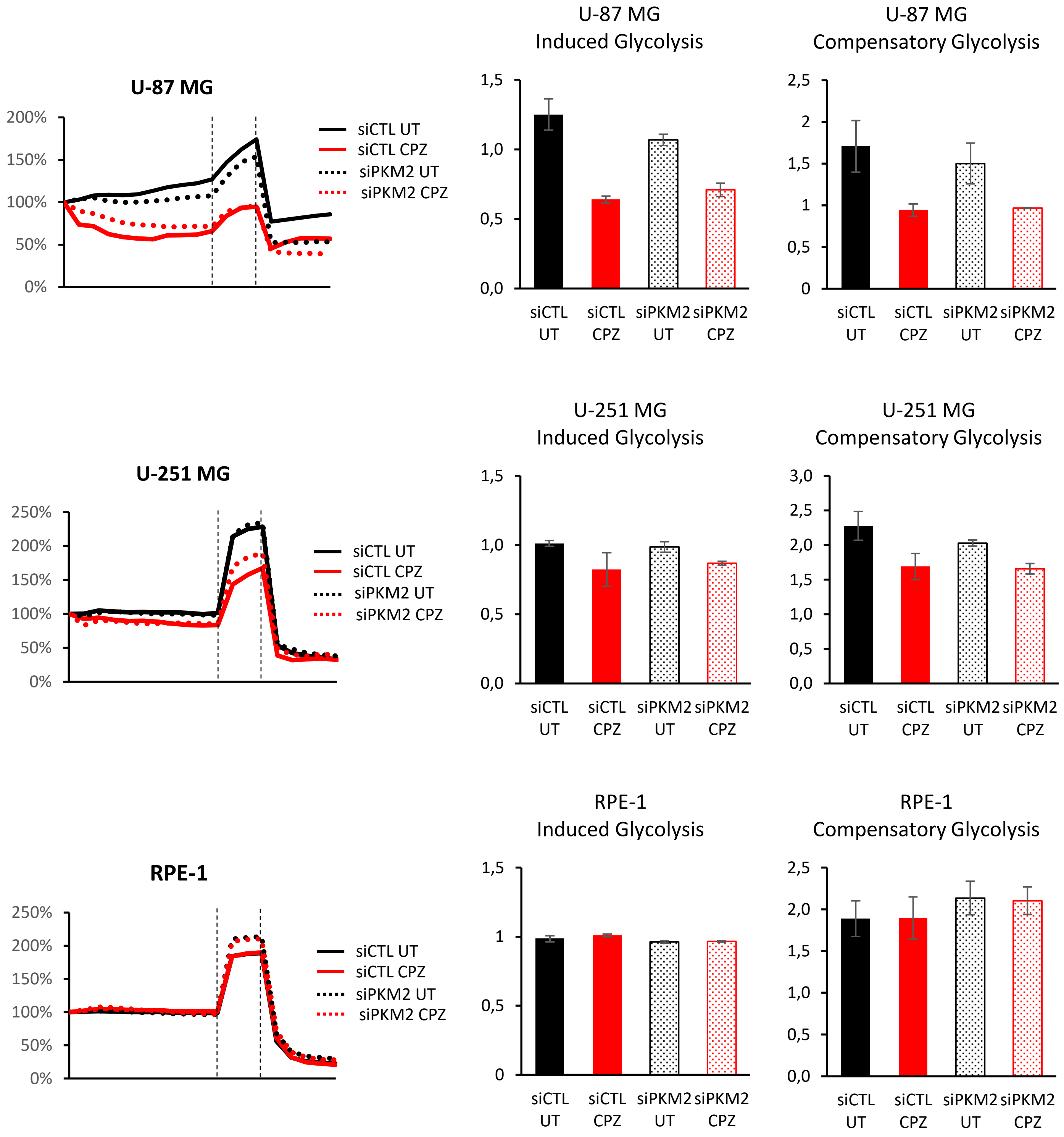

Effect of siRNA silencing on extracellular lactate concentration. Left: graphs from Seahorse output data were averaged from two triplicate experiments, and glycoPER values were normalized to 100% at the time of drug (or mock) injection. siCTL-transfected cells extracellular lactate production is represented by solid lines, while siPKM2-transfected cells are represented by dotted lines, black for untreated cells and red for CPZ-treated ones. The histograms represent induced (central histogram) and compensatory (left histogram) glycolysis, averaged from the two triplicate output data. Solid bars refer to siCTL-transfected cells, while dotted bars refer to siPKM2-transfected cells, treated with mock (black) or CPZ (red), respectively.

Figure S4

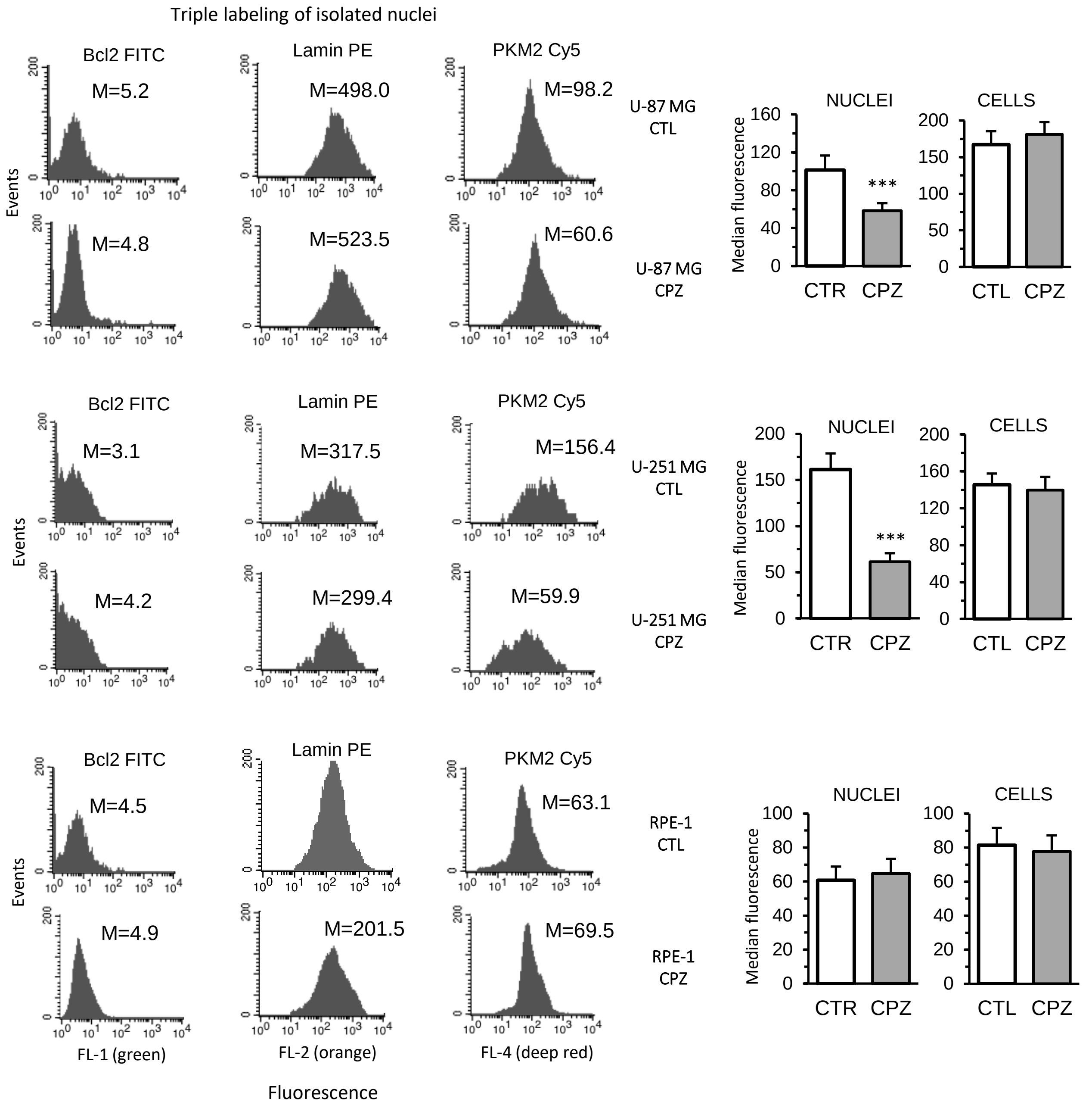

CPZ decreases nuclear PKM2 localization in GBM cells: Nuclei were isolated from control and CPZ-treated cells as described under Materials and Methods, immunolabeled and then analyzed on a fluorescent cytometer. On the left, representative graphs show cytofluorimetric expression levels of Bcl2 (as a cytosolic marker), Lamin A/C (as positive control for nuclei) and PKM2 in nuclei from anchorage-dependent cells. Results from three independent experiments are reported as median fluorescence ± SD in the histograms on the right of the panel. Asterisks denote statistical significance (***p<0.001).

Figure S5

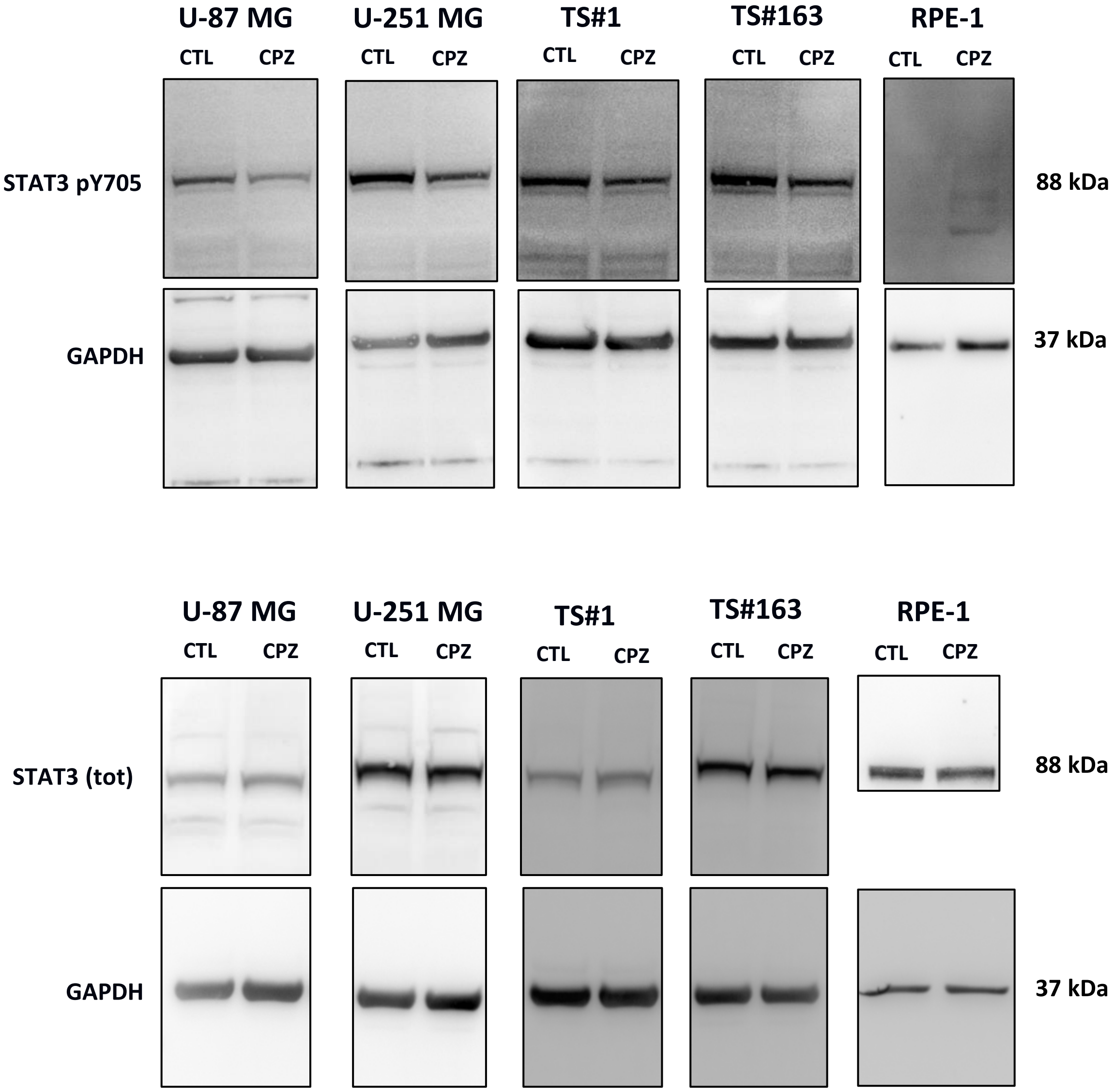

Figure S5. Representative western blot analyses of STAT3 pY705 and total STAT3 in control and CPZ-treated cells. GAPDH is shown for normalization purposes

Figure S6

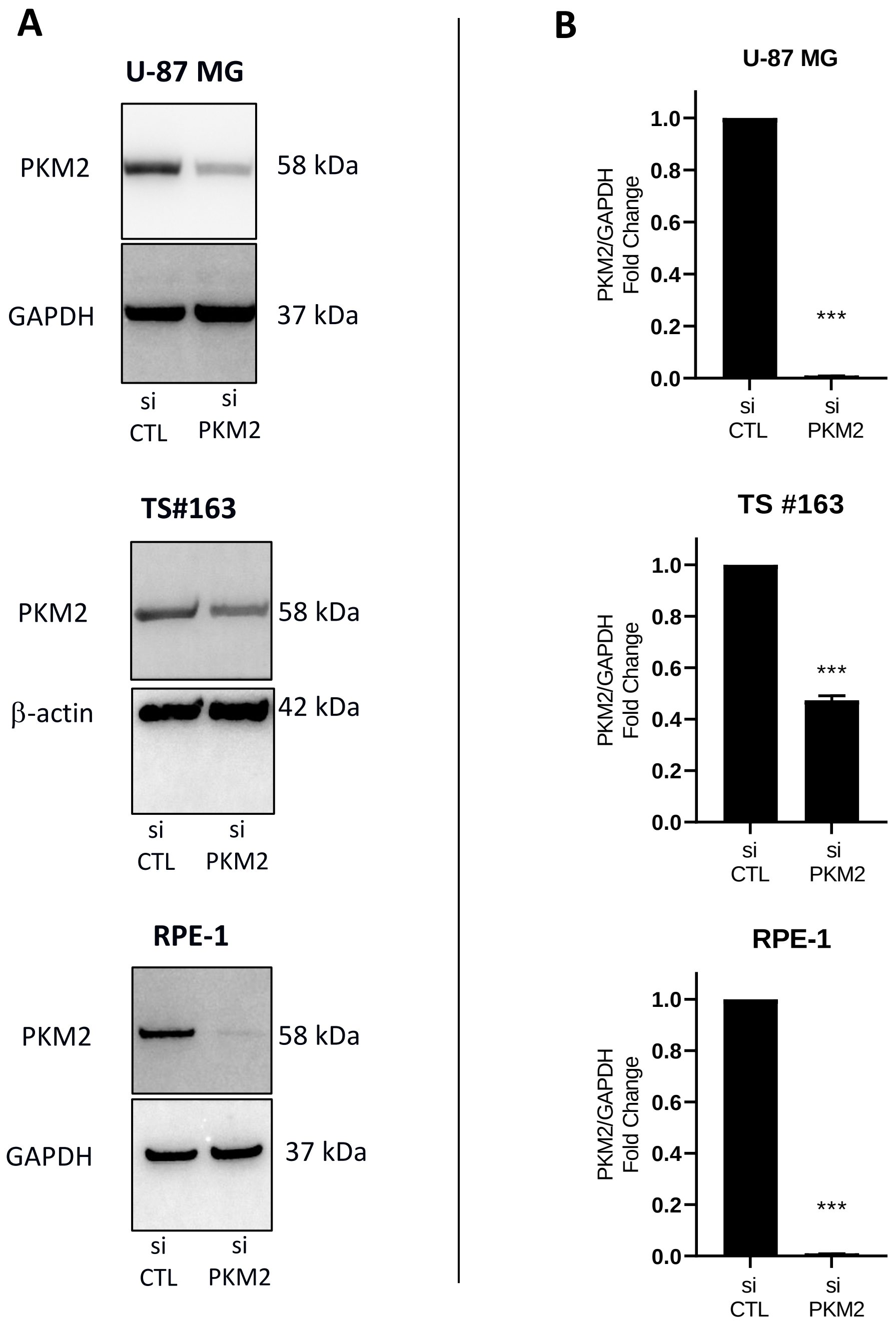

PKM2 silencing in U-87 MG GBM cells, TS#163 neurospheres and RPE-1 non-cancer neuroepithelial cells. **A**. Western blots: representative images show that specific siRNA-PKM2 silencing elicited an apparent reduction in PKM2 protein expression in all cell lines, when compared with siRNA-Control. GAPDH or β-actin are shown for normalization purposes. **B**. RT-qPCR: specific siRNA-PKM2 silencing elicited an apparent reduction in PKM2 mRNA amount in all cell lines; histograms show a quantitative and statistical analysis of PKM2 mRNA relative amount normalized against GAPDH mRNA. Asterisks denote statistical significance (***$p<0.001$).
