# Supplementary material for: Chlorpromazine affects glioblastoma bioenergetics by interfering with pyruvate kinase M2
Source: Cell Death Dis. 2023 Dec 13;14(12):821. doi: 10.1038/s41419-023-06353-3 (PMC10719363; doi:10.1038/s41419-023-06353-3)
Supplement: Supplementary file 3 — Original Data File [file 41419_2023_6353_MOESM3_ESM.pdf]

Figure 5

Western blotting U-87 MG

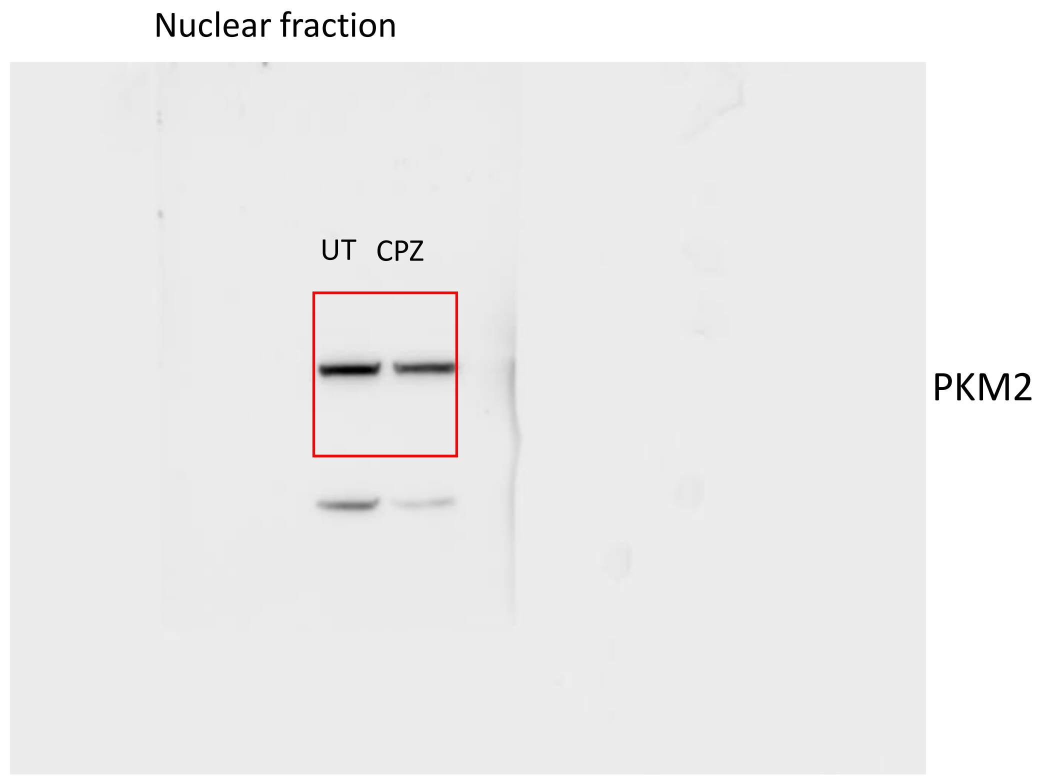

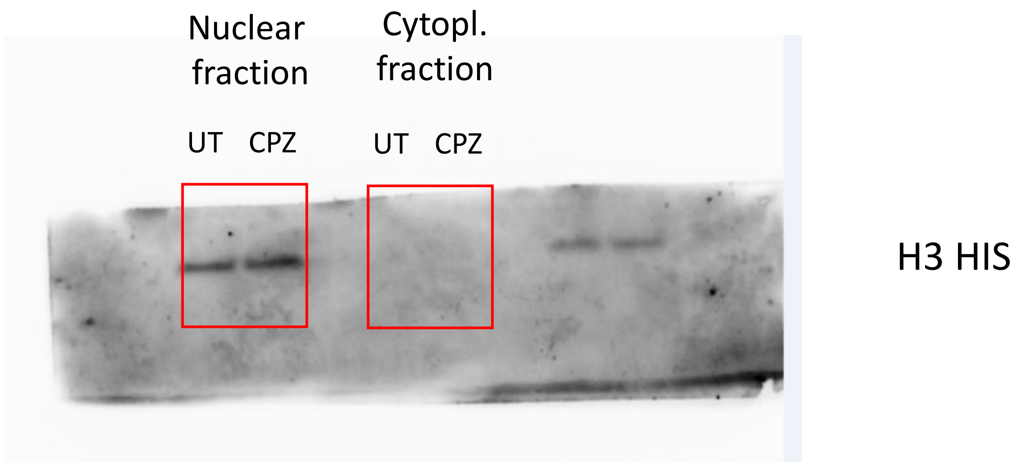

Figure 5

Western blotting U-87 MG

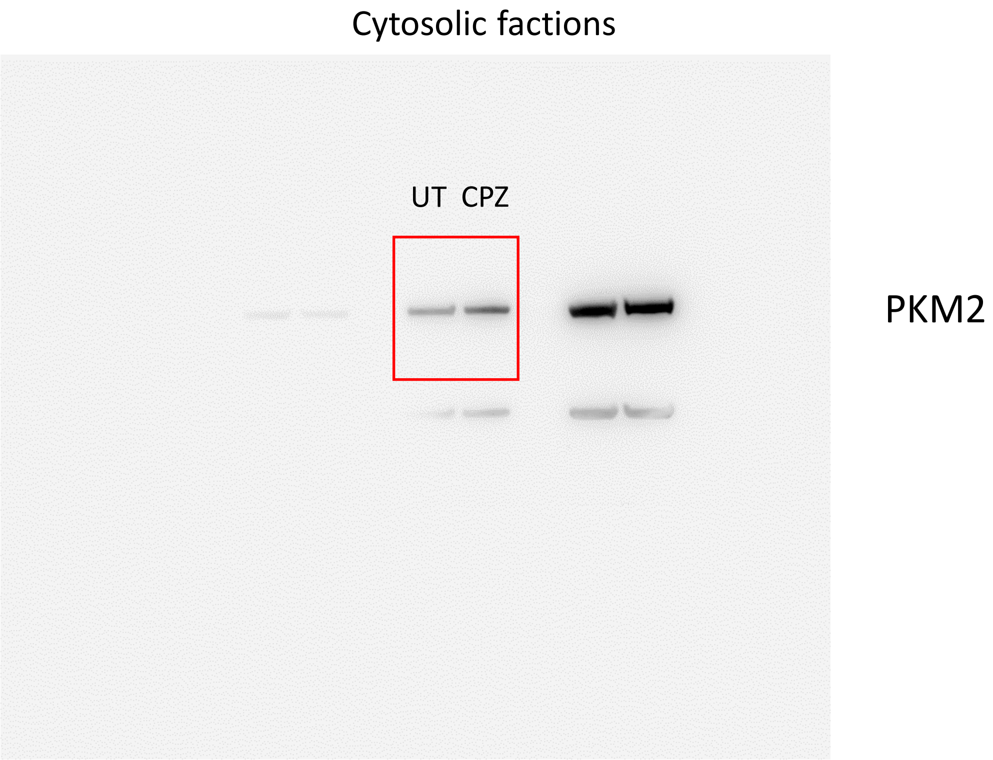

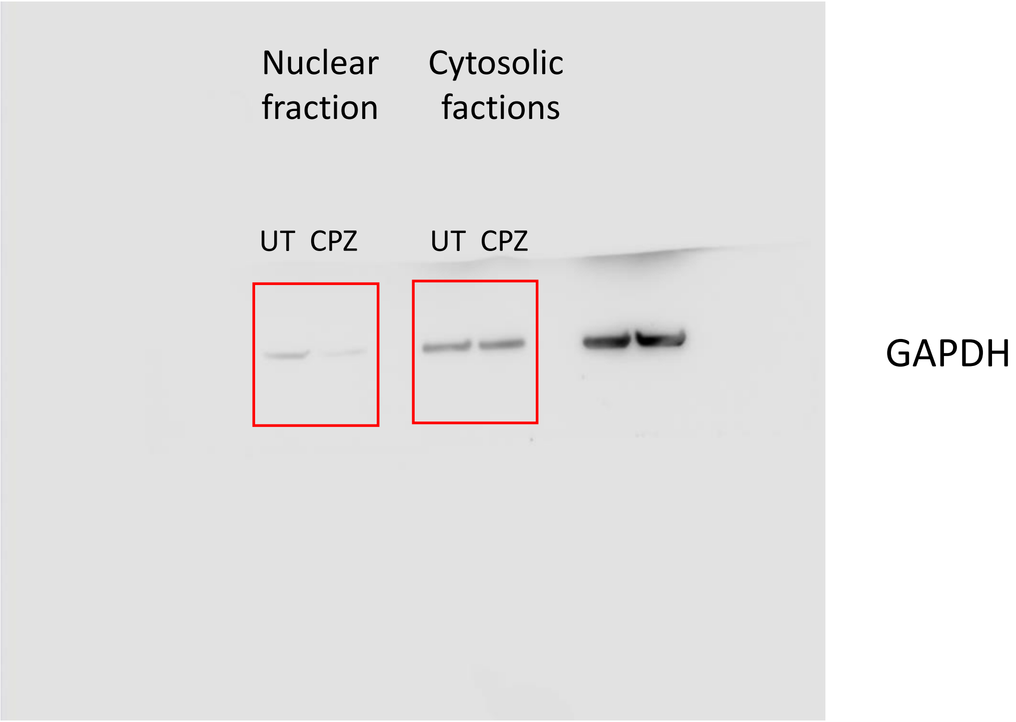

Figure 5

Western blotting U251-MG

Nuclear fraction

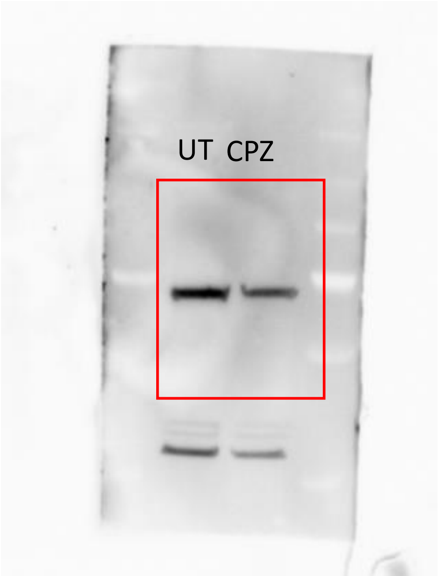

PKM2

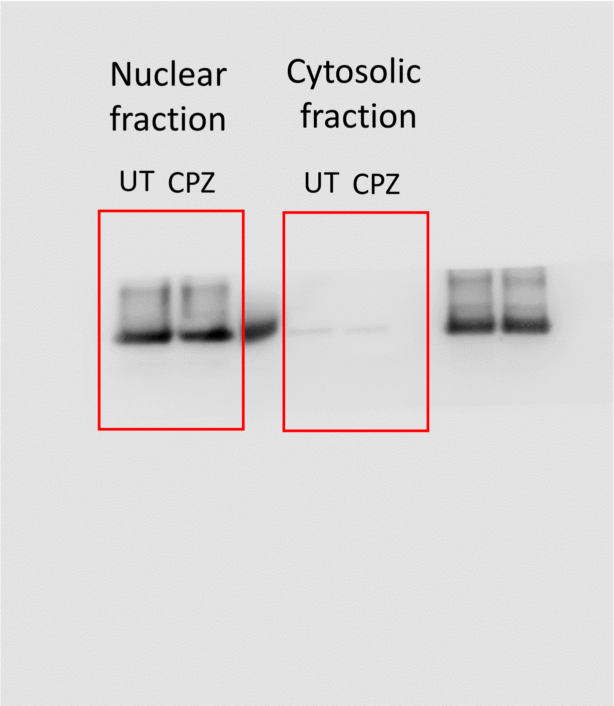

H3 HIS

Figure 5 Western blotting U251-MG

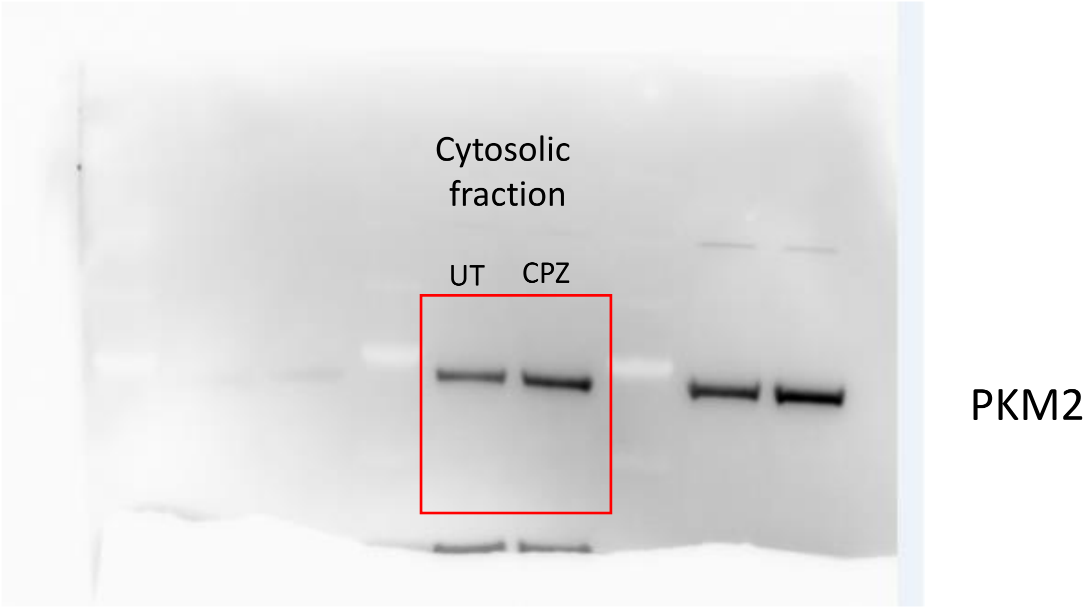

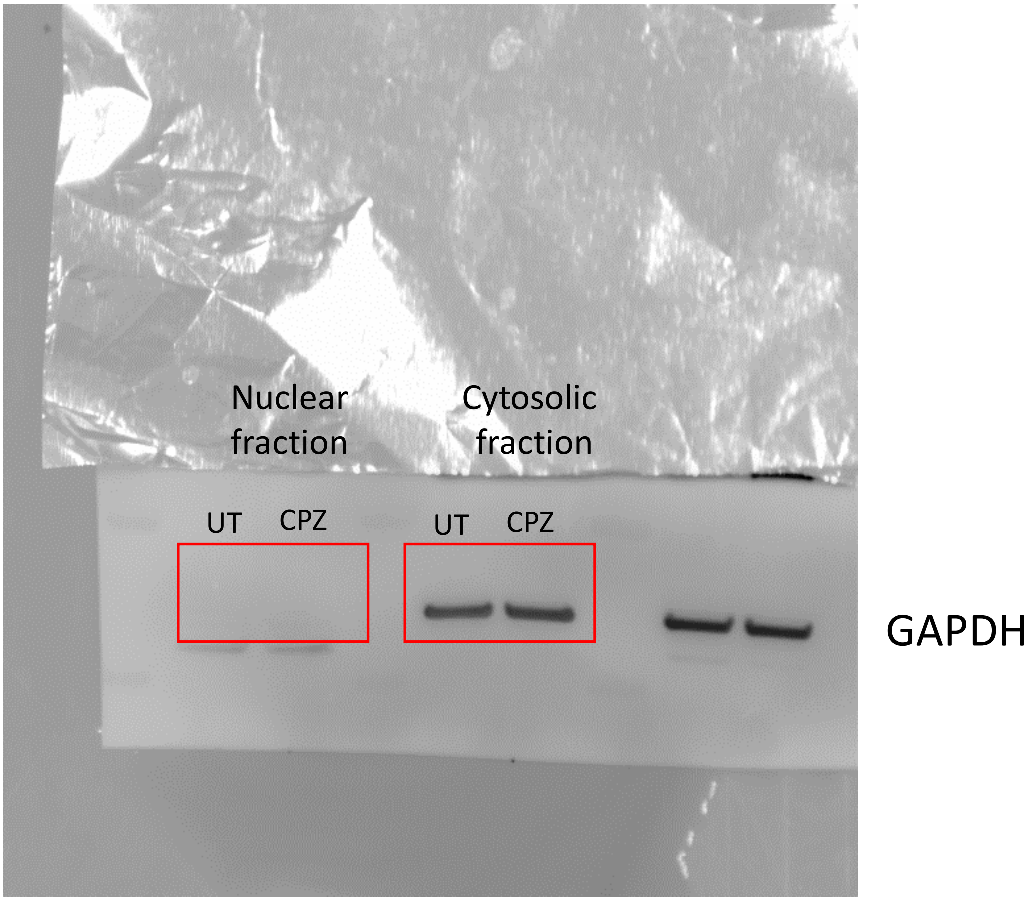

Figure 5

Western blotting RPE-1

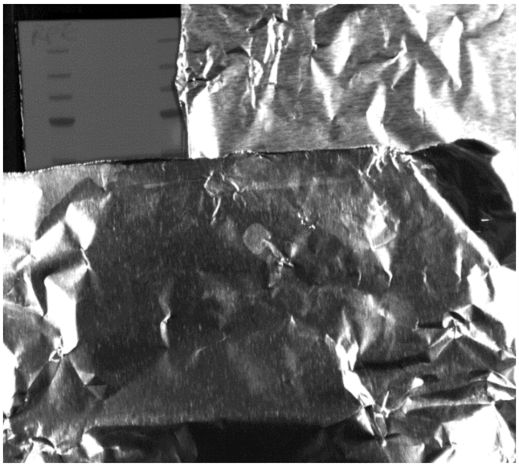

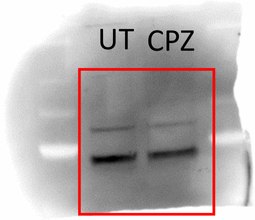

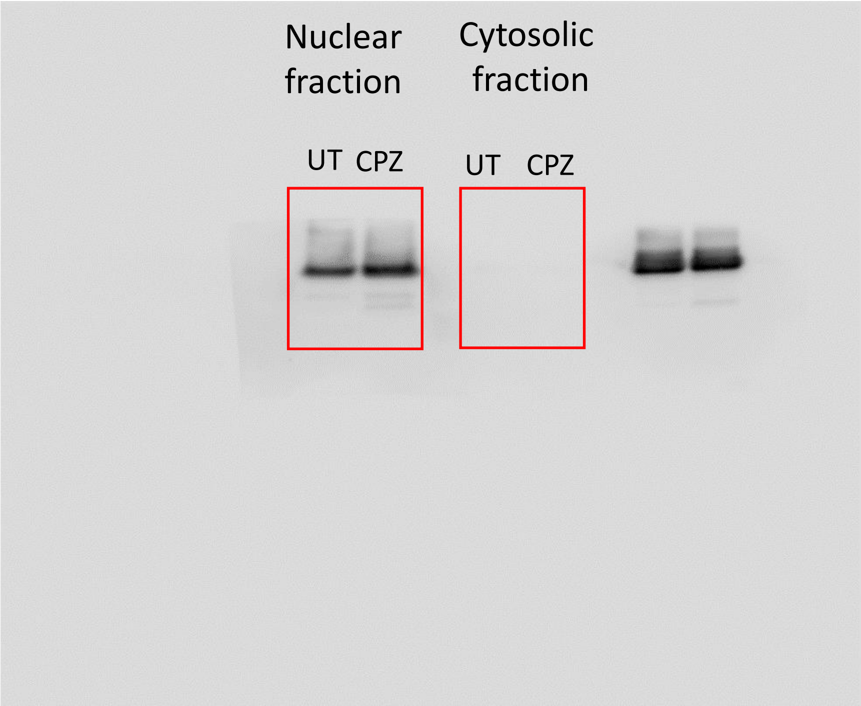

Figure 5

Western blotting RPE-1

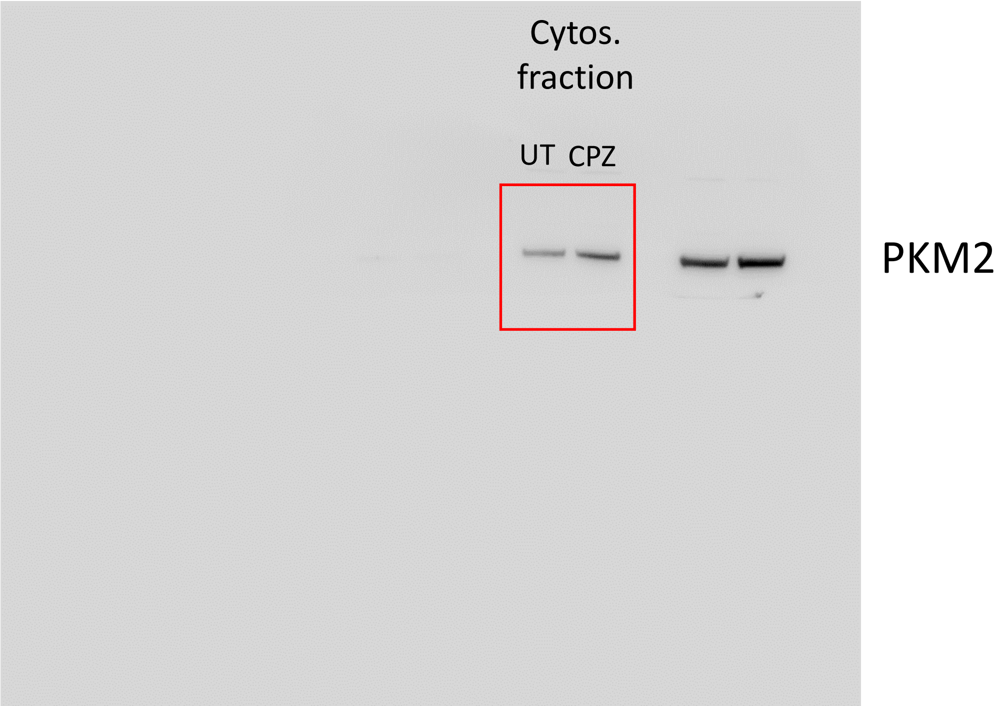

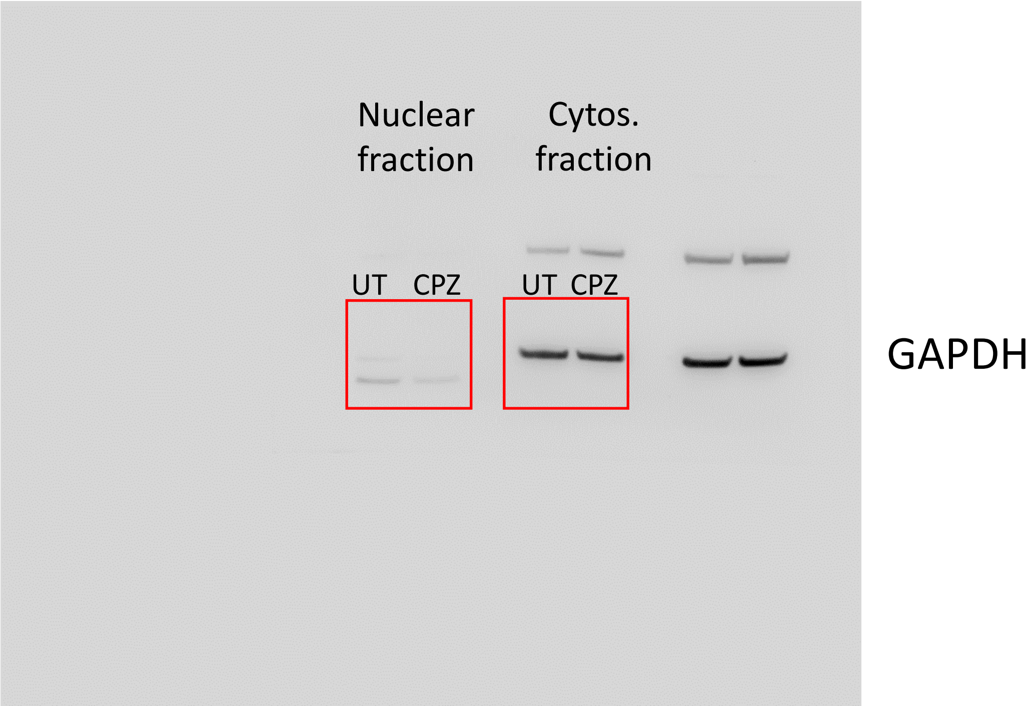

Figure 5 Western blotting TS #1

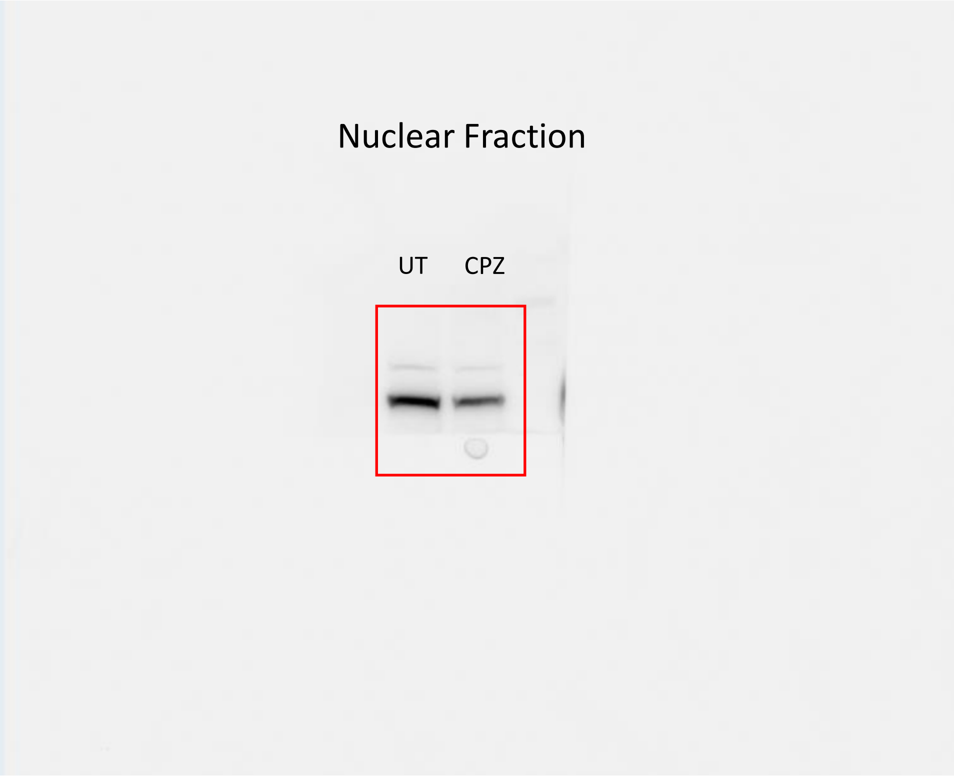

PKM2

Nuclear fraction

Cytosolic fraction

UT CPZ UT CPZ

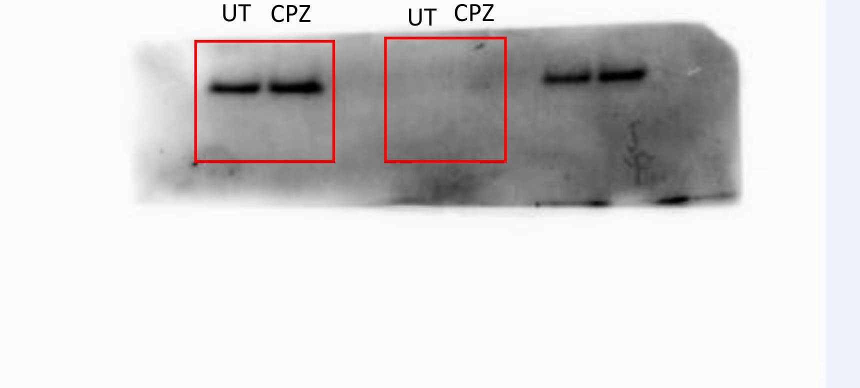

H3 His

Figure 5

Western blotting TS #1

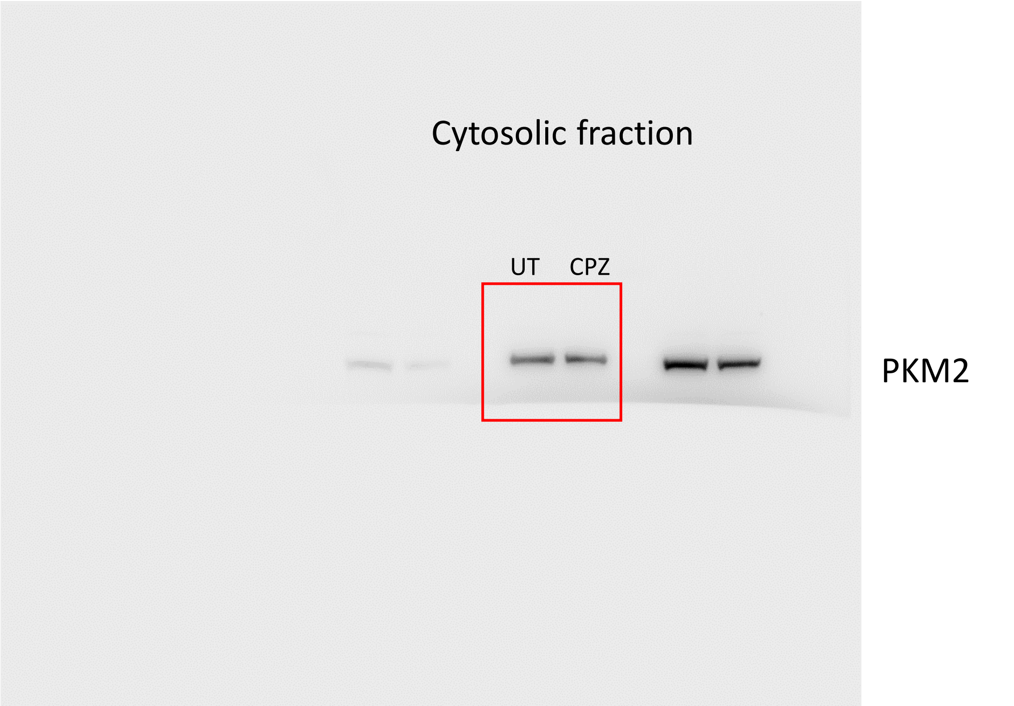

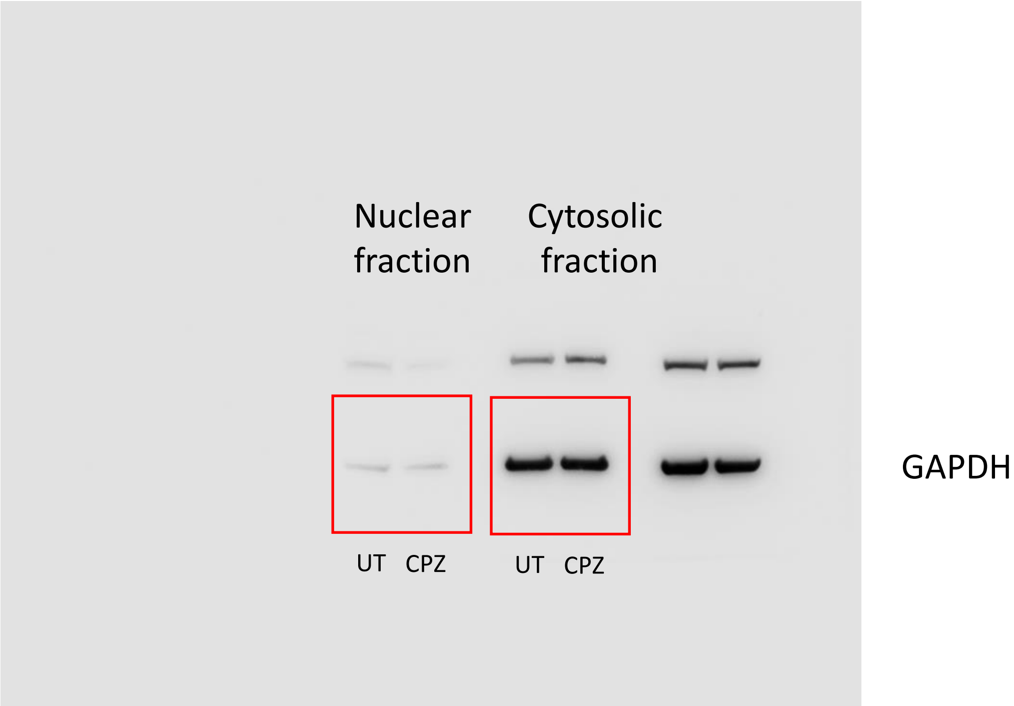

Figure 5

Western blotting TS #163

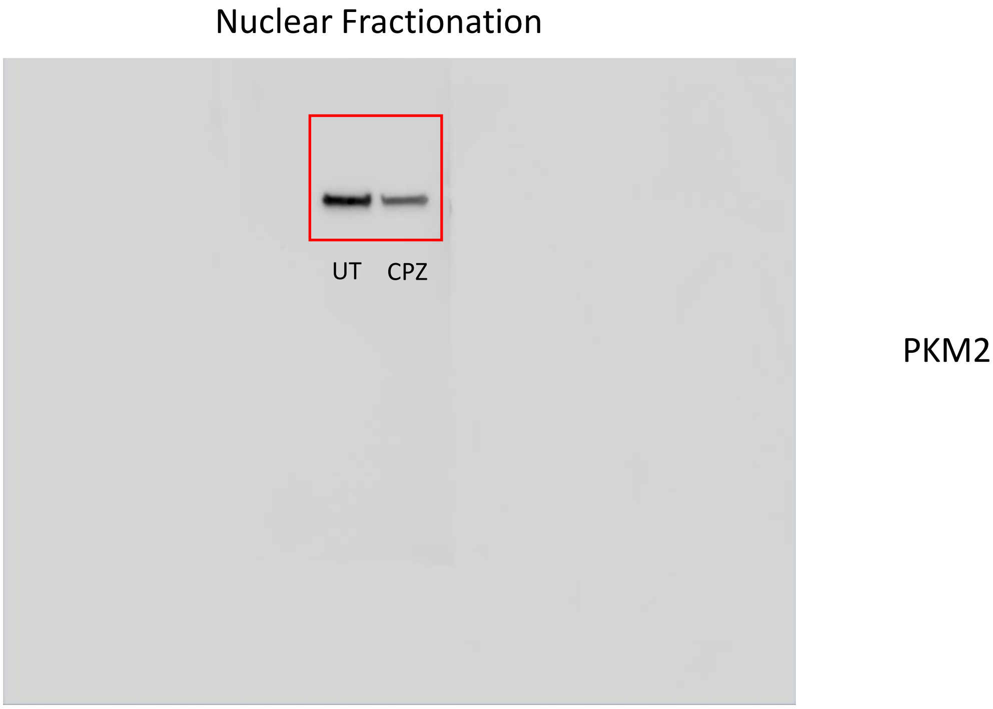

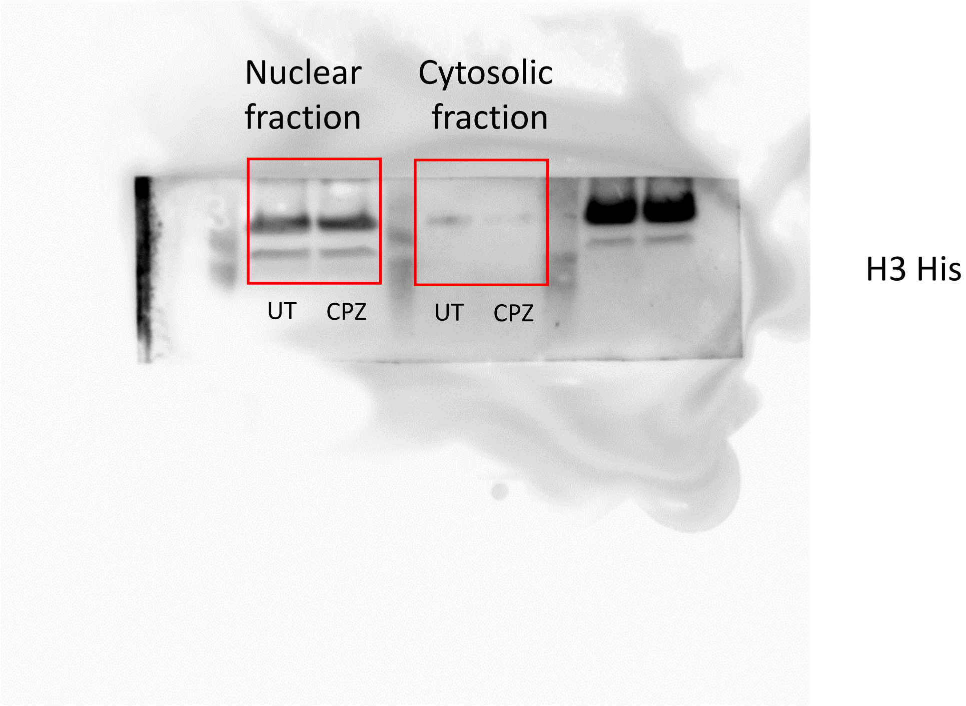

Figure 5

Western blotting TS #163

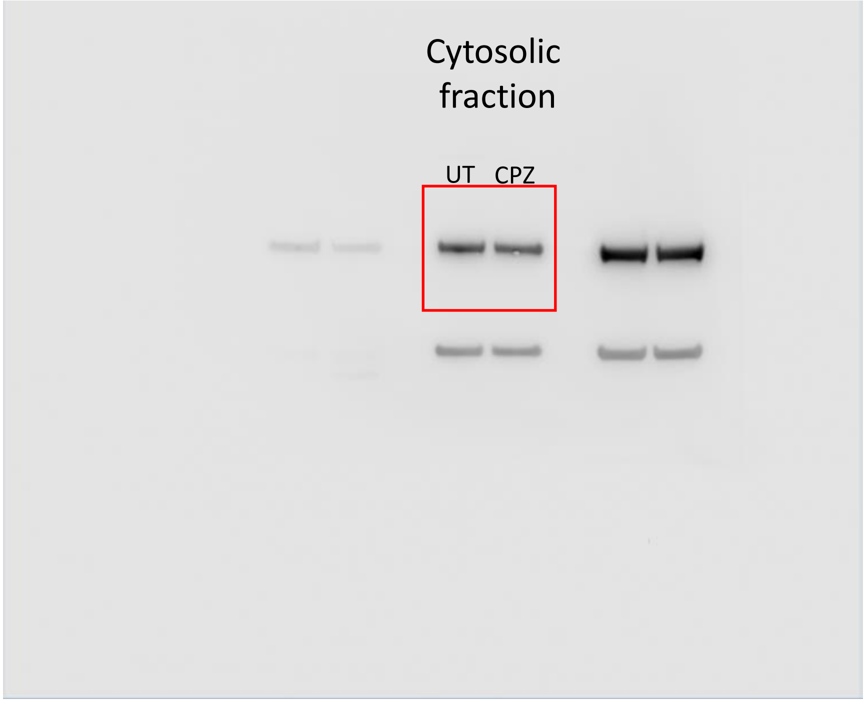

PKM2

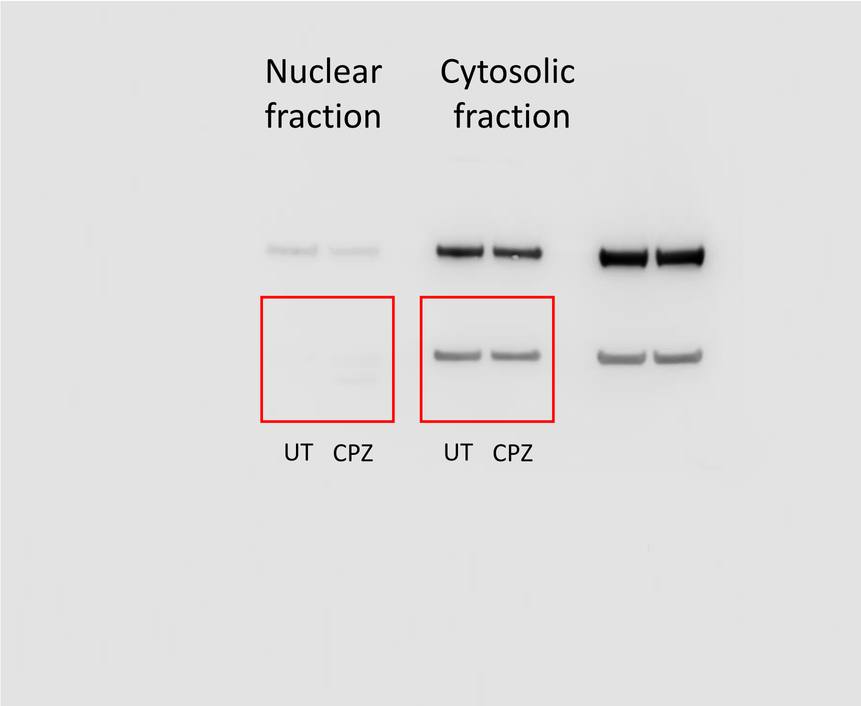

GAPDH

Figure S2

Western blotting siPKM2 U-87 MG (Seahorse)

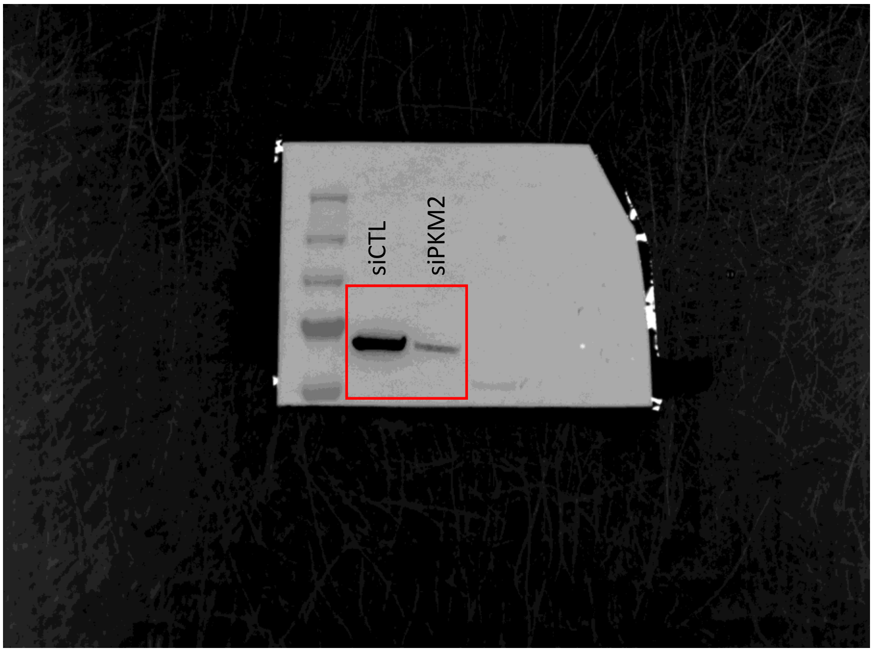

PKM2

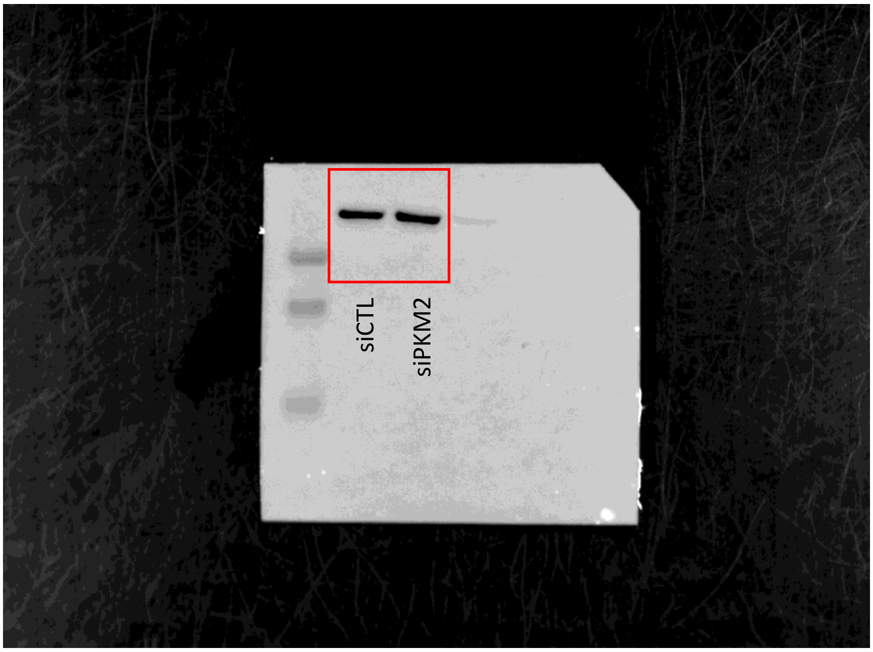

GAPDH

Figure S2

Western blotting siPKM2 U-251 MG (Seahorse)

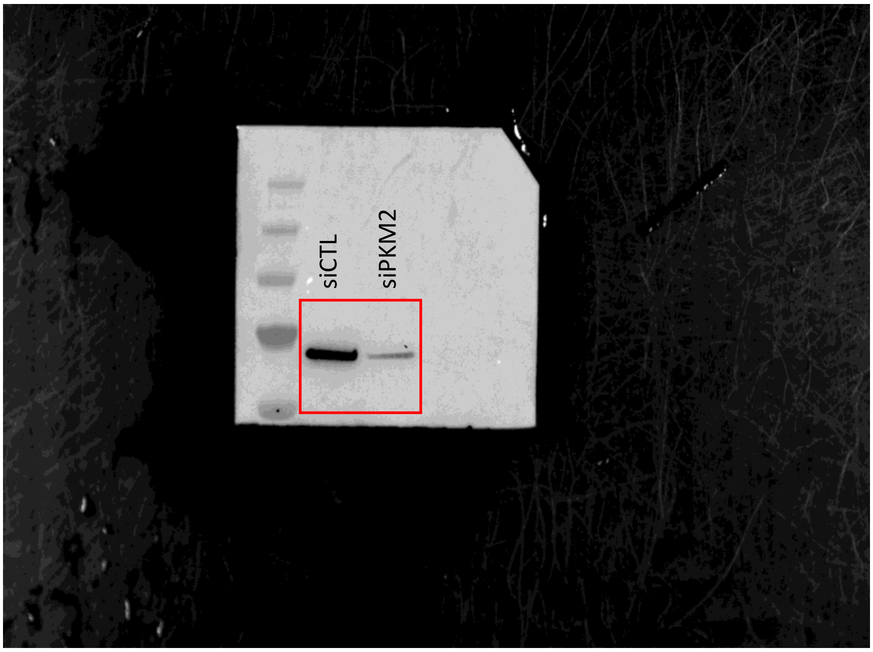

PKM2

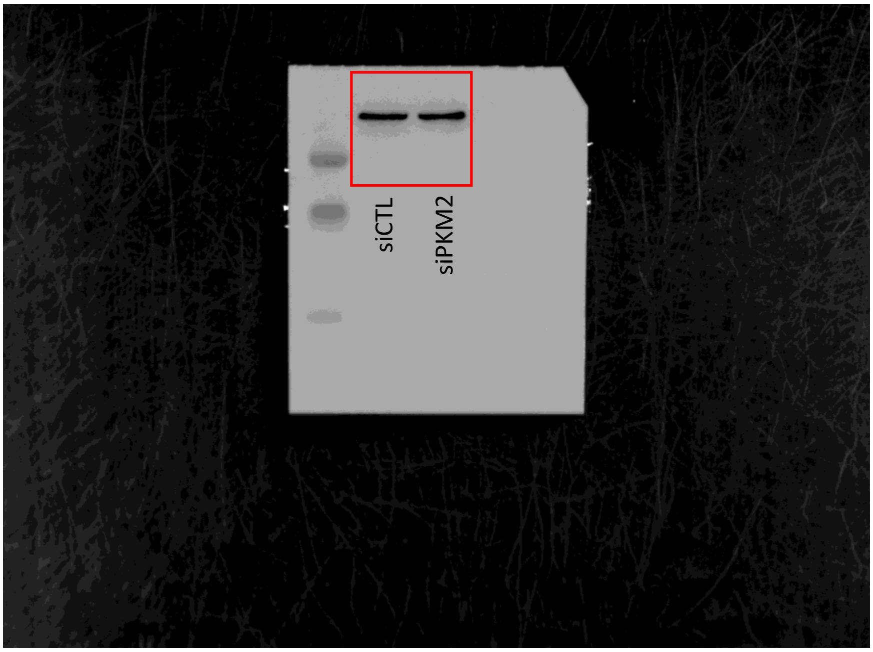

GAPDH

Figure S2

Western blotting siPKM2 RPE-1 (Seahorse)

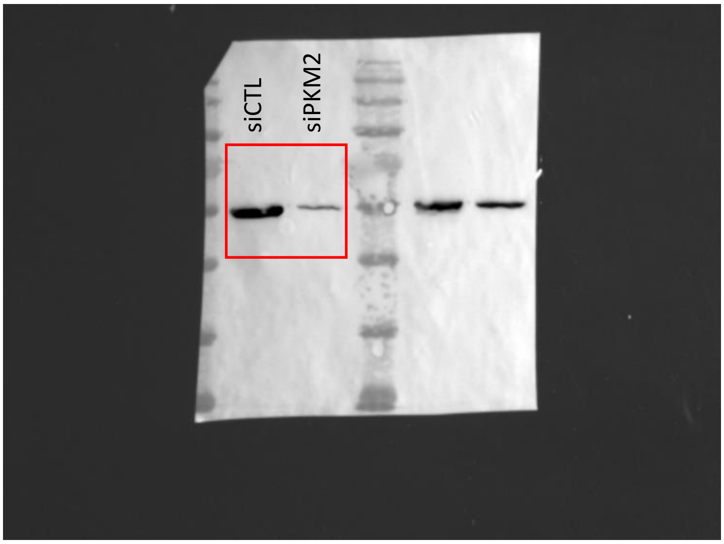

PKM2

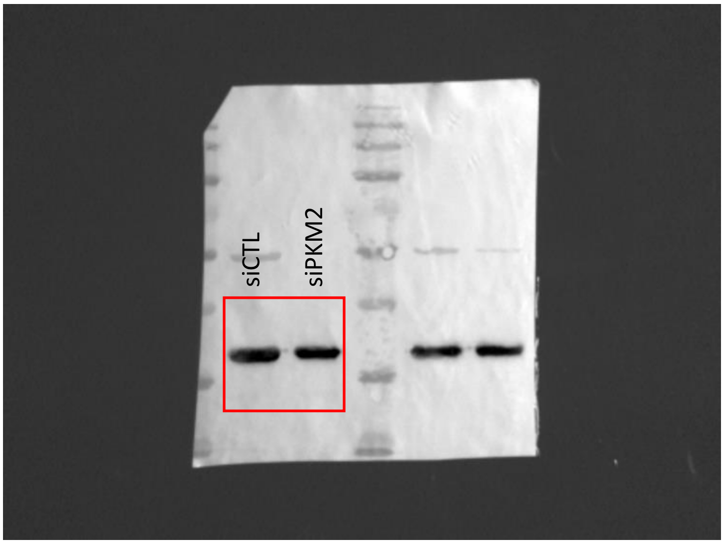

GAPDH

Figure S5

Western Blots STAT3 pY705 U-87 MG and U251 MG

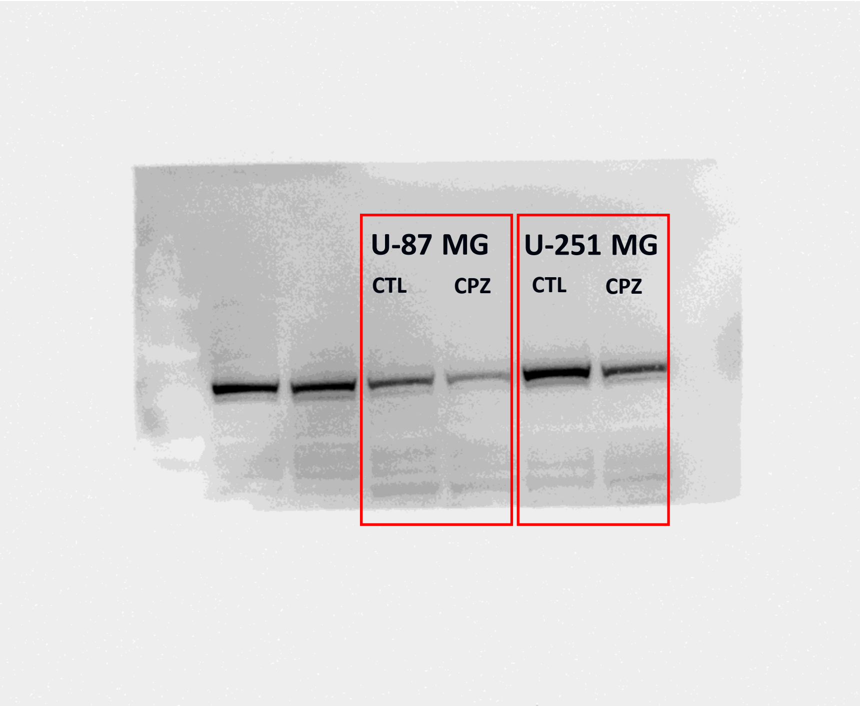

STAT3 pY705

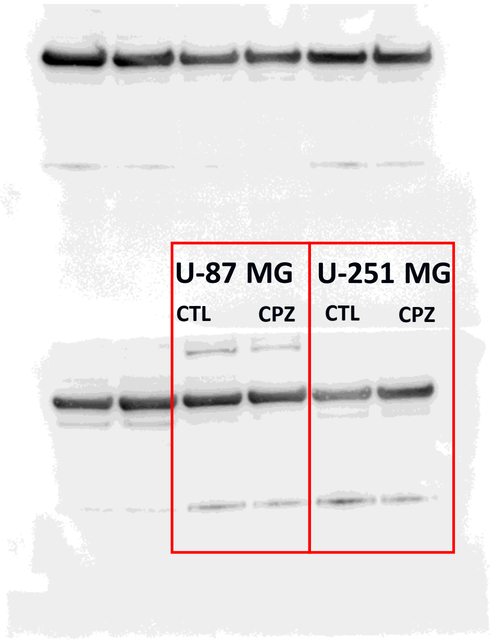

GAPDH

Figure S5

Western Blots STAT3 pY705 TS#1 and TS#163

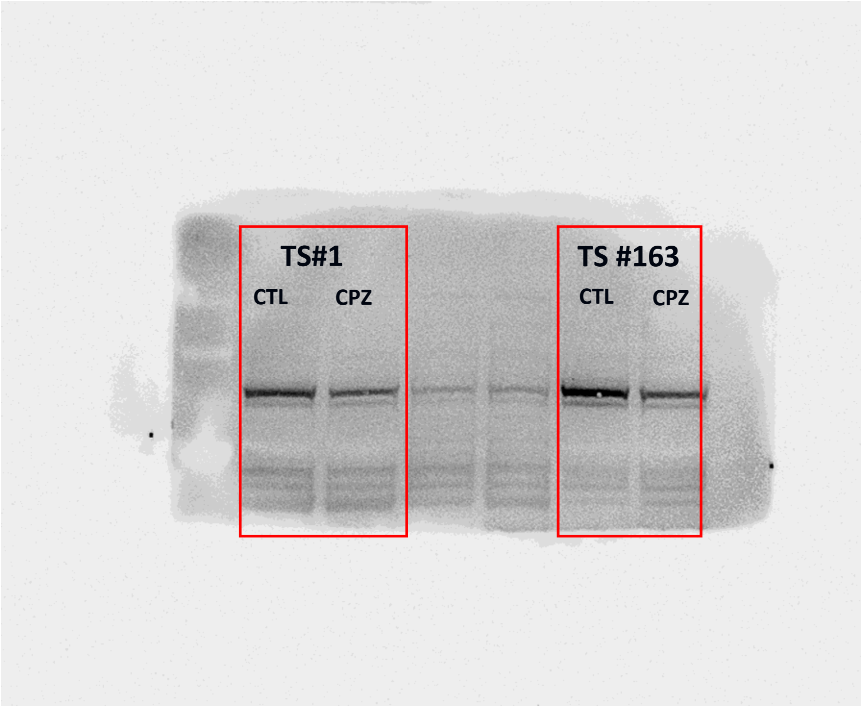

STAT3 pY705

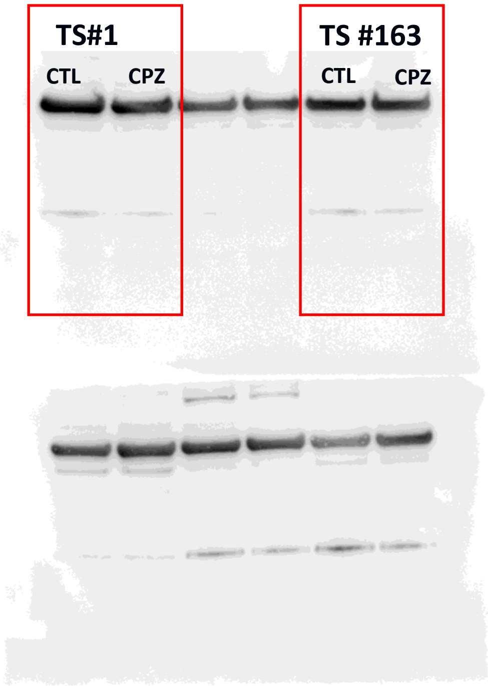

GAPDH

Figure S5

Western Blots STAT3 pY705 RPE-1

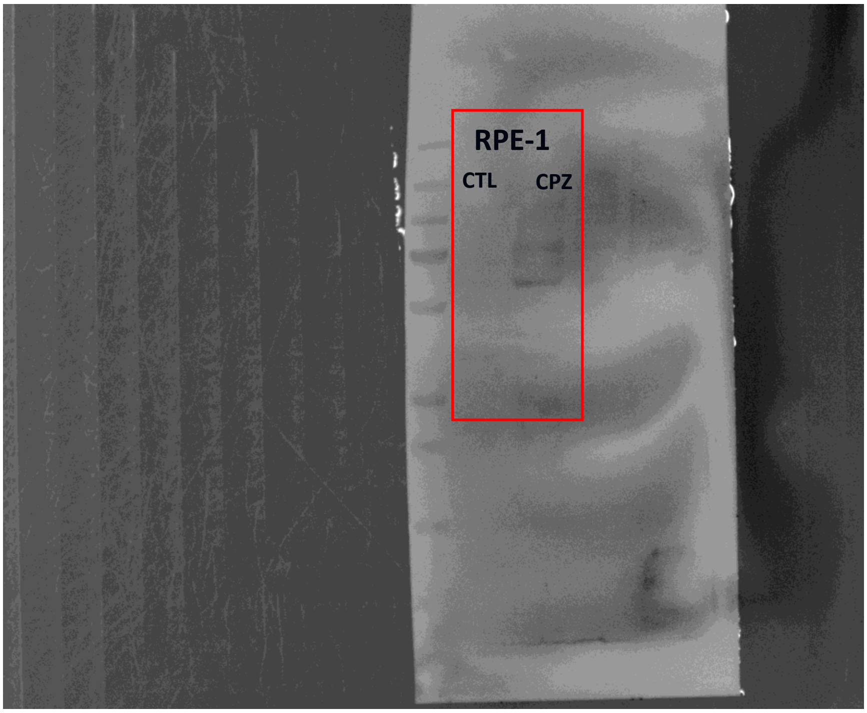

STAT3 pY705

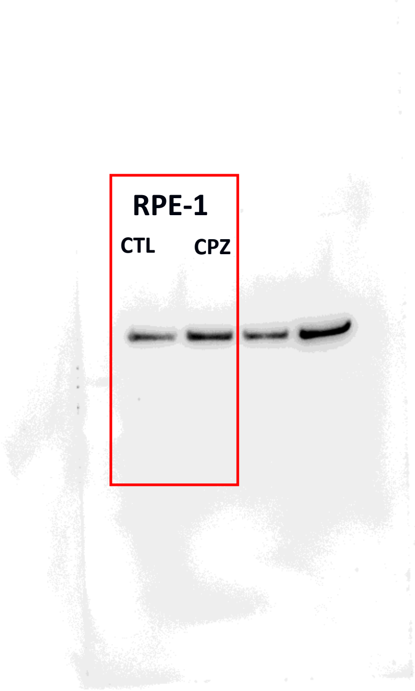

GAPDH

Figure S5

Western Blots STAT3 (tot) U-87 MG and U251 MG

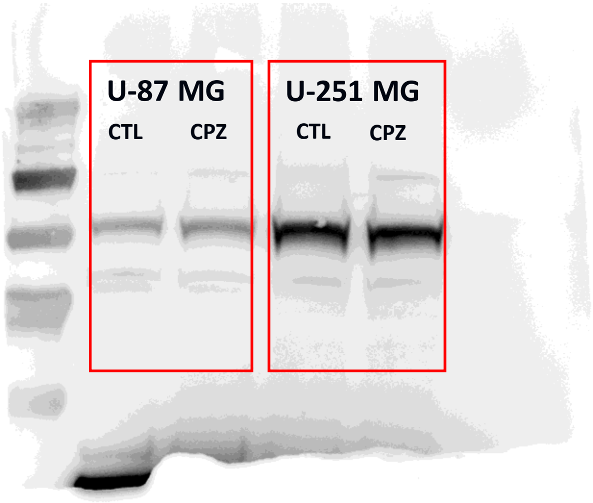

STAT3 (tot)

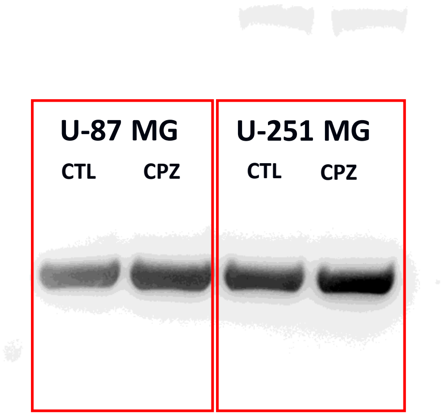

GAPDH

Figure S5

Western Blots STAT3 (tot) TS#1 and TS#163

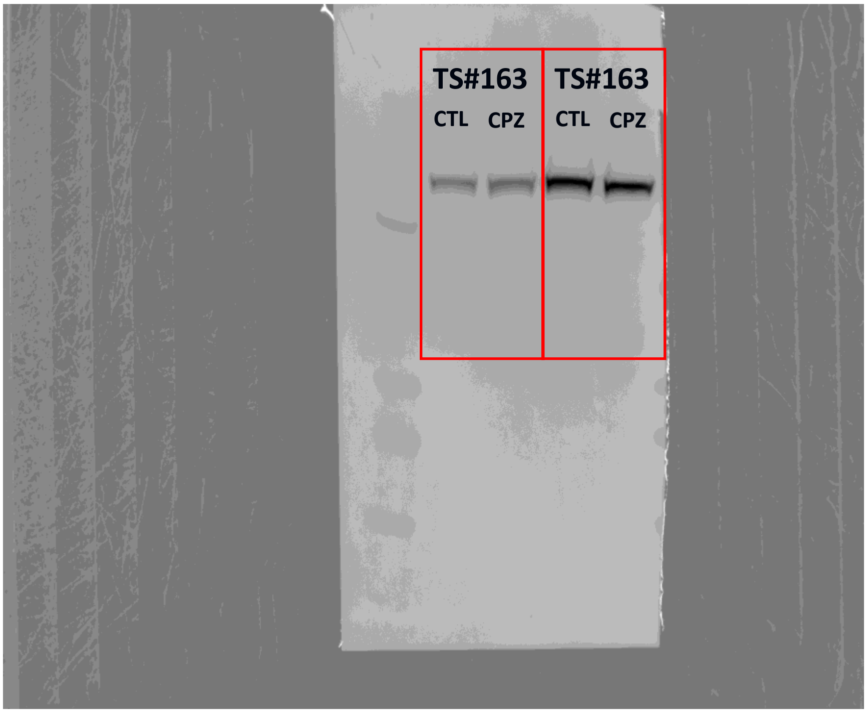

STAT3 (tot)

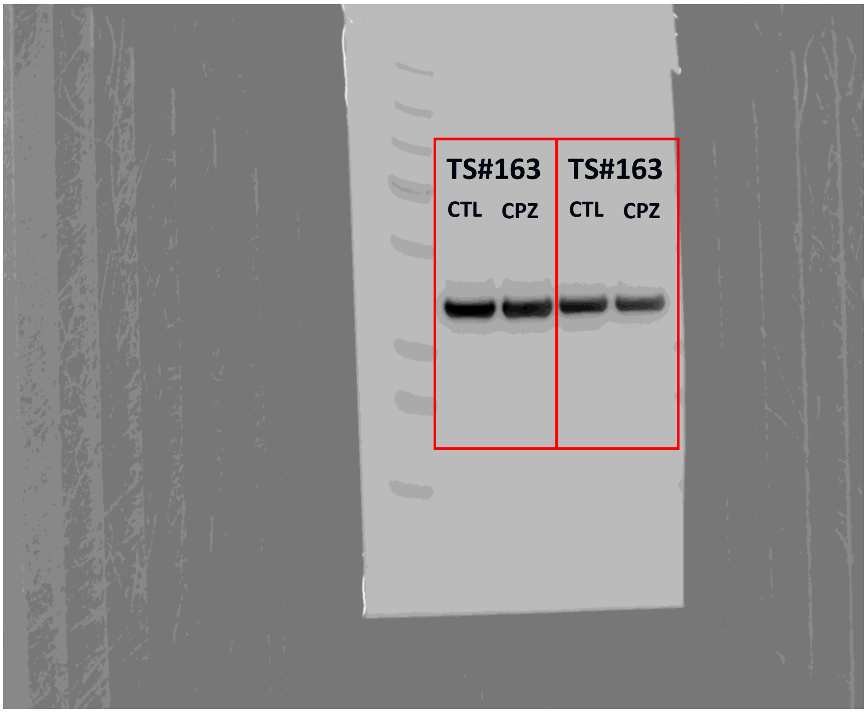

GAPDH

Figure S5

Western Blots STAT3 (tot) RPE-1

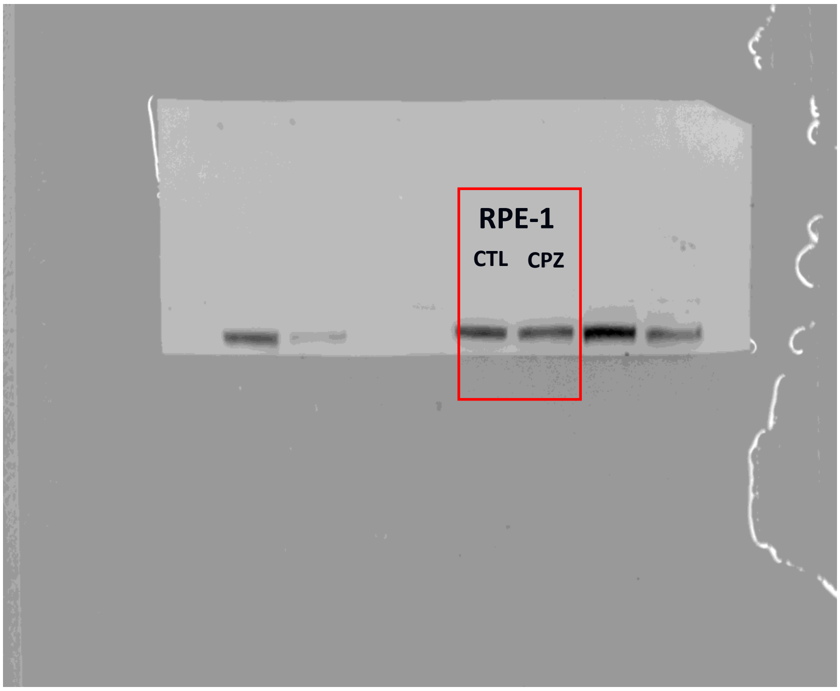

STAT3 (tot)

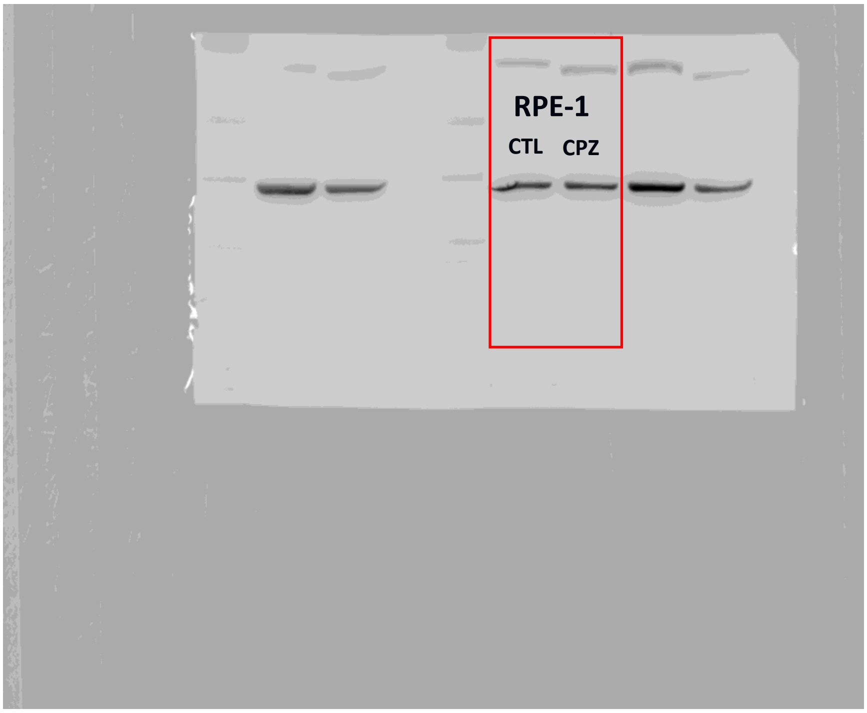

GAPDH

Figure S6

Western Blots siPKM2 U-87 MG

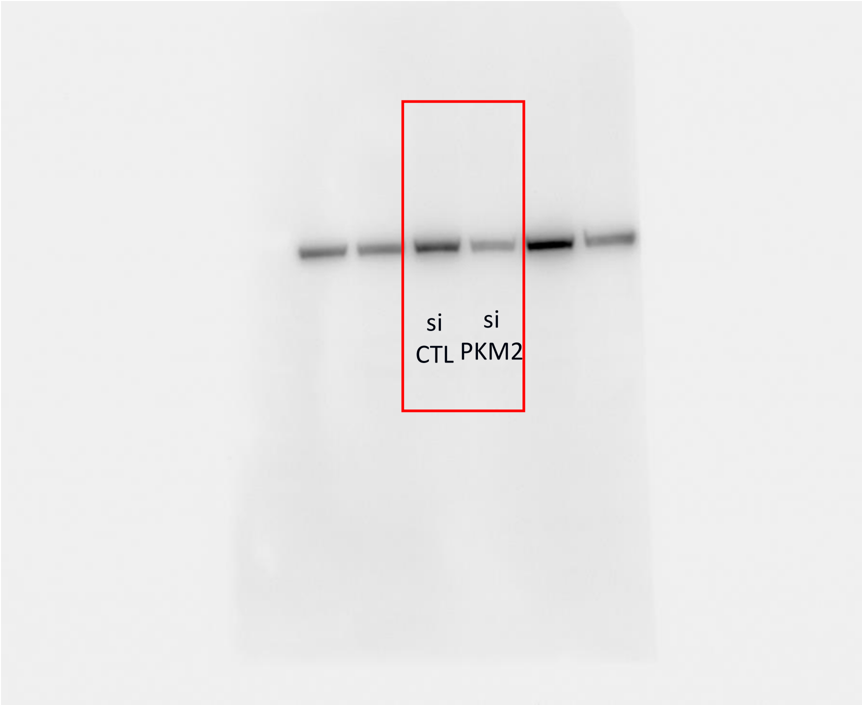

PKM2

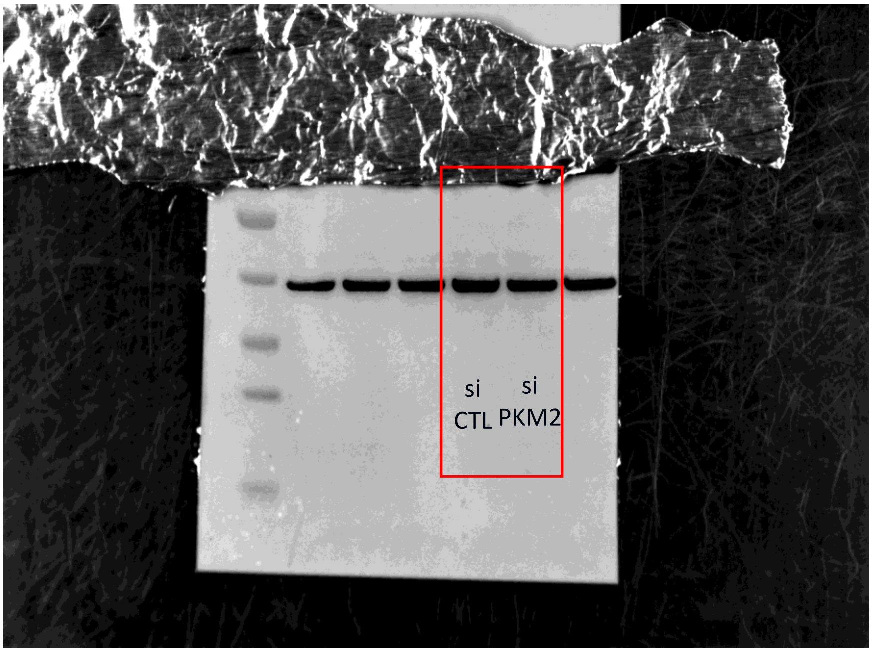

GAPDH

Figure S6

Western Blots siPKM2 #163

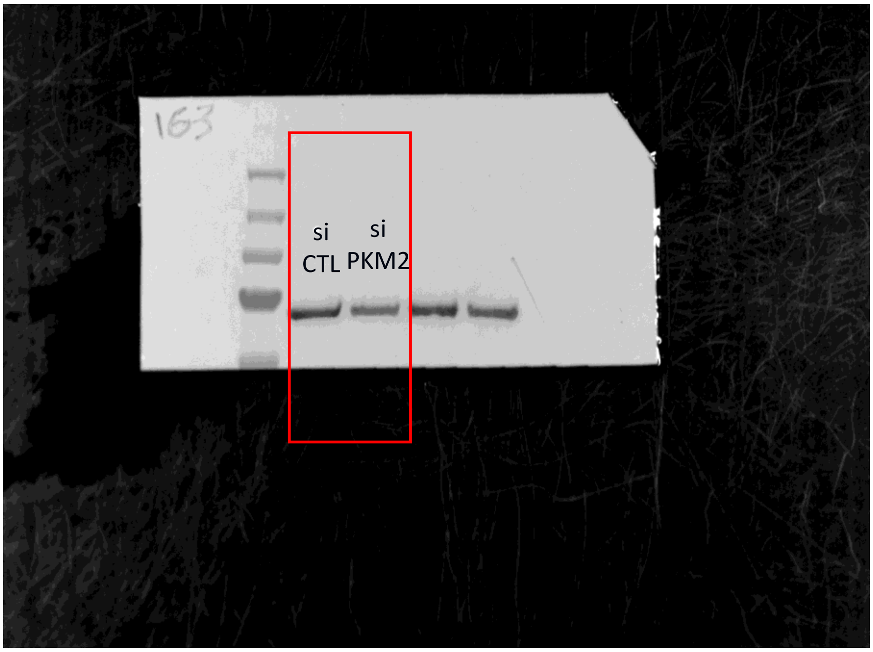

PKM2

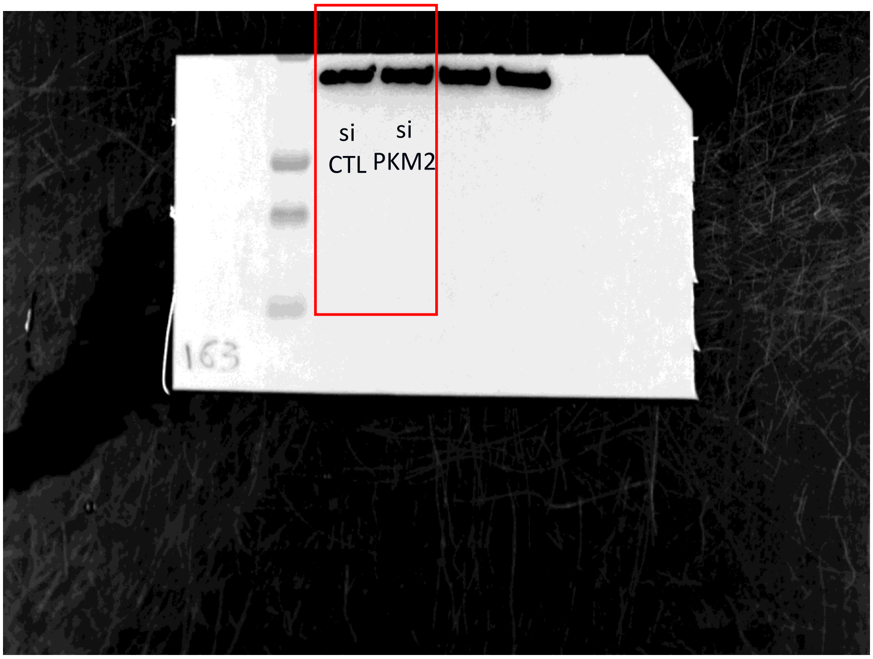

β-actin

Figure S6

Western Blots siPKM2 RPE-1

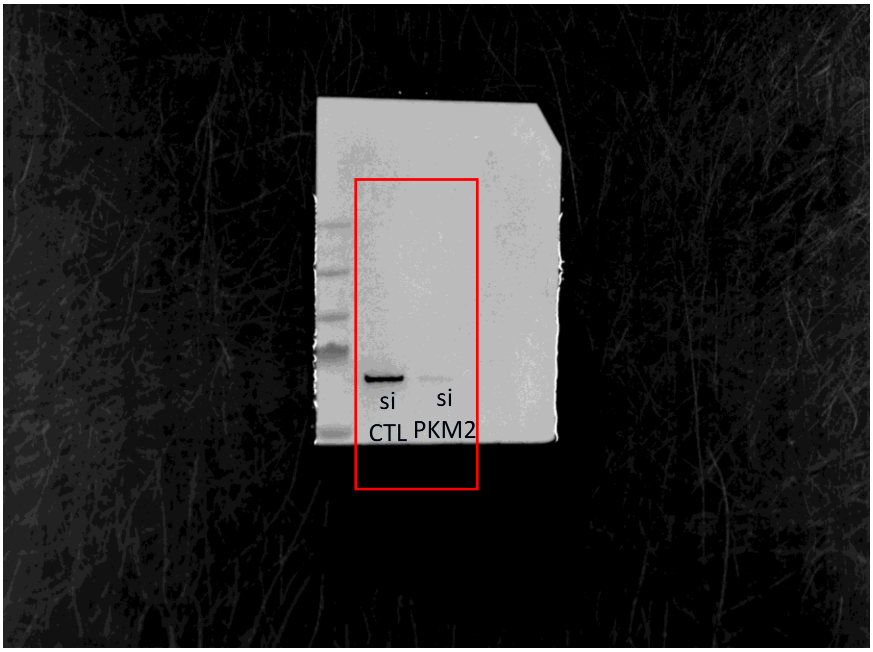

PKM2

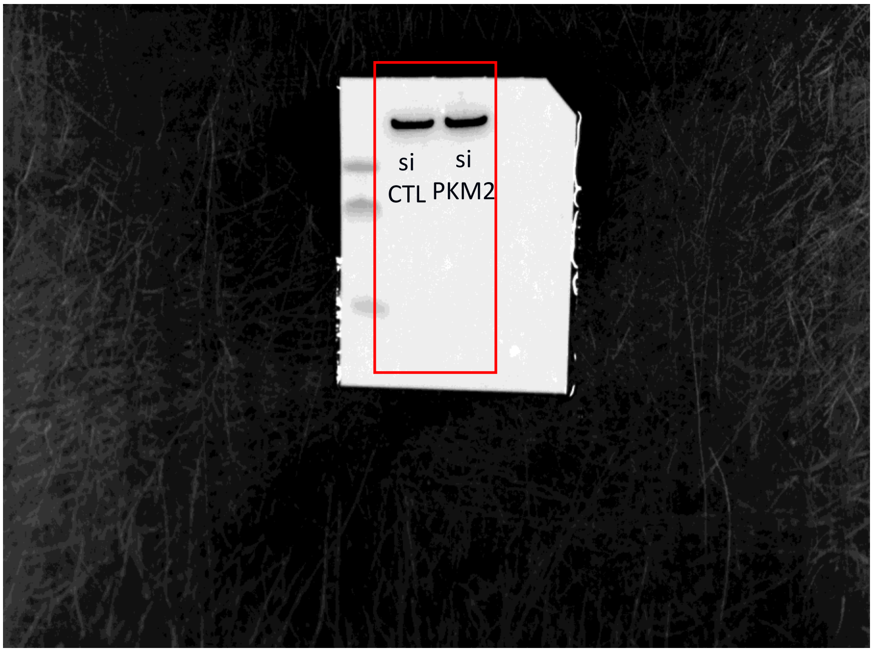

GAPDH
